# Cellular and Molecular Mechanisms of Micro- and Nanoplastics Driving Adverse Human Health Effects

**DOI:** 10.3390/toxics13110921

**Published:** 2025-10-28

**Authors:** Antonio F. Hernández, Marina Lacasaña, Aristidis M. Tsatsakis, Anca Oana Docea

**Affiliations:** 1 Department of Legal Medicine and Toxicology, School of Medicine, University of Granada, 18016 Granada, Spain; 2 Health Research Institute of Granada (Instituto Biosanitario de Granada, Ibs.GRANADA), 18012 Granada, Spain; marina.lacasana.easp@juntadeandalucia.es; 3 Consortium for Biomedical Research in Epidemiology and Public Health (CIBERESP), 28029 Madrid, Spain; 4 Andalusian School of Public Health (EASP), 18011 Granada, Spain; 5 Andalusian Health and Environment Observatory (OSMAN), 18011 Granada, Spain; 6 Center of Toxicology & Science Applications, Medical School, University of Crete, 71003 Heraklion, Greece; tsatsaka@uoc.gr; 7 Human Development and Health Science Faculty, Universidad ECOTEC, Km 13.5 Samborondón, Samborondon 092302, Ecuador; 8 Biomedical Science and Technology Park, Sechenov I.M., First State Medical University, 119991 Moscow, Russia; 9 Department of Toxicology, University of Medicine and Pharmacy of Craiova, 200349 Craiova, Romania; daoana00@gmail.com

**Keywords:** micro- and nanoplastics (MNPs), systemic toxicity, target-organ toxicity, environmental pollutants, environmental health risks

## Abstract

Micro- and nanoplastics (MNPs) are increasingly recognized as emerging contaminants of concern for human health. Their small size, diverse composition, and reactive surface enable interactions with biological barriers and cellular systems. This comprehensive narrative review synthesizes and critically evaluates current evidence on the mechanistic effects of MNPs in humans and experimental models. Systemic mechanisms, including oxidative stress, inflammation, barrier disruption, and immune dysregulation, may underlie reported adverse effects in the gastrointestinal tract, cardiovascular, nervous and reproductive systems, as well as the placenta. Omics studies further reveal alterations in metabolic and stress-response pathways, providing systems-level insights and candidate biomarkers. Human data remain limited to biomonitoring studies, and causality has not yet been established. Toxicological data, though informative, often rely on pristine particles and high-dose, short-term exposures that exceed environmental estimates, highlighting the need for chronic, low-dose models. Major challenges include difficulties in detecting and quantifying MNPs in tissues, limited attribution of effects to polymers versus additives or adsorbed contaminants, and lack of standardized characterization and reporting. Emerging advances, such as reference materials, omics profiling, and organ-on-chip technologies, offer opportunities to close these gaps. Overall, the available data suggest biologically plausible pathways for health risks, but methodological refinement and harmonized research strategies are essential for robust human health assessment.

## 1. Introduction

The widespread use of plastics over the past century has led to a global pollution crisis, with plastic waste accumulating in terrestrial, freshwater, and marine environments. Among the most concerning byproducts are microplastics (MPs; <5 mm) and nanoplastics (NPs; <1 µm), which arise either from intentional manufacturing or from the degradation of larger plastic materials. These particles have been detected in oceans, soil, drinking water, air, and in human matrices such as blood, lungs, placenta, and stool, raising pressing questions about their potential impact on human health [1,2,3,4].

Micro- and nanoplastics (MNPs) are not chemically inert; they may contain additives (e.g., phthalates, bisphenol A, flame retardants) and can adsorb and transport environmental pollutants such as persistent organic pollutants (POPs) and heavy metals. Consequently, they may act not only as physical stressors, but also as chemical carriers within biological systems, thereby amplifying toxic effects, a phenomenon known as the “Trojan Horse” effect [5,6,7].

While initial studies focused mainly on ecological toxicity in aquatic organisms, recent findings from mammalian models and limited human data indicate bioaccumulation and potential toxic effects across various organ systems. MNPs have been shown to cross critical physiological barriers such as the intestinal epithelium, placental barrier, and possibly the blood–brain barrier (BBB), depending on their size and surface characteristics [3,8].

Despite the growing number of toxicological studies on MNPs, much of the current literature remains fragmented, relies on in vitro or high-dose animal models, and lacks standardization in terms of particle type, size distribution, and exposure protocols. Furthermore, human health risk assessment is constrained by limited epidemiological data, inconsistent detection methods, and the absence of established exposure thresholds. As a result, regulatory bodies such as the World Health Organization (WHO) and European Food Safety Authority (EFSA) acknowledge the plausibility of health concerns but highlight the uncertainty and data gaps that preclude drawing definitive conclusions [9,10].

This comprehensive narrative review aims to synthesize and critically evaluate current mechanistic evidence on the effects of MNPs in humans and experimental models, while identifying methodological gaps and proposing recommendations for future research and regulation. We examine the physicochemical properties relevant to toxicity, cellular uptake and translocation across biological barriers, and the cellular and molecular mechanisms underlying systemic and organ-specific effects. Understanding these processes is essential for establishing causality and informing health risk assessment. The review was developed using an expert-driven synthesis approach, supported by targeted PubMed/MEDLINE searches and citation tracking, with additional methodological details provided in the Supplementary Materials.

## 2. Physicochemical Properties of MNPs and Relevance to Toxicity

Physicochemical characteristics, including particle size, shape, surface charge (also known as zeta potential), polymer type, chemical additives, and adsorbed environmental pollutants, critically determine the biological behavior and toxicity of MNPs [11]. Among these, particle size plays a particularly important role: polystyrene nanoparticles (PS-NP, <200 nm) exhibit significantly higher rates of cellular internalization, tissue penetration and subcellular organelle localization than larger particles [12].

Particle shape is another determinant of biological interaction. While many experimental studies use spherical particles, irregular fragments and fibrous MNPs are increasingly recognized in environmental samples. Fibers can deposit deeply in the lung, particularly within alveolar regions, where their elongated geometry hinders clearance by macrophages and promotes persistent inflammation [13]. In contrast, spherical and irregular fragments are more readily internalized via endocytosis, whereas fibers may induce frustrated phagocytosis, amplifying oxidative and inflammatory responses [14]. These differences highlight the need to account for realistic environmental morphologies when assessing human health risks.

Surface chemistry and charge also modulate MNPs’ bio-interactions by influencing the composition and conformation of the protein corona, a dynamic layer of biomolecules that adsorbs onto particle surfaces upon contact with biological fluids. This bio-corona alters cellular recognition, trafficking, and immune reactivity [15,16]. For example, amine-modified polystyrene (PS-NH_2_) nanoparticles provoke stronger cytotoxic and inflammatory reactions than their neutral (PS) or carboxylated (PS-COOH) counterparts [11,12,17]. Positively charged PS-NP are particularly potent, as they destabilize cell membranes, increase permeability and promote mitochondrial damage, reactive oxygen species (ROS) generation, and apoptotic signaling [18].

In addition to their inherent physical and chemical properties, MNPs often act as vectors for leachable additives such as phthalates, bisphenol A (BPA), and flame retardants. These chemicals can be released following particle internalization or interaction with physiological fluids, further exacerbating MNPs’ toxicity [19].

Altogether, these multifactorial characteristics, spanning particle morphology, surface charge, and chemical payload, contribute to the diverse and complex toxicological outcomes associated with MNPs. This underscores the critical need for detailed physicochemical characterization and standardized particle profiling in experimental designs assessing MNP safety.

## 3. Cellular Uptake and Translocation Across Biological Barriers

MNPs enter cells through various pathways, including clathrin- and caveolin-mediated endocytosis, macropinocytosis, and passive diffusion across lipid membranes. Due to their extremely small size, NP can penetrate cellular membranes and disrupt fundamental processes [20]. For instance, PS-NPs smaller than 100 nm are readily internalized by HeLa cells via active endocytic mechanisms, whereas larger particles (>250 nm) tend to remain extracellular and exhibit reduced cytotoxicity [21].

**Figure 1:**
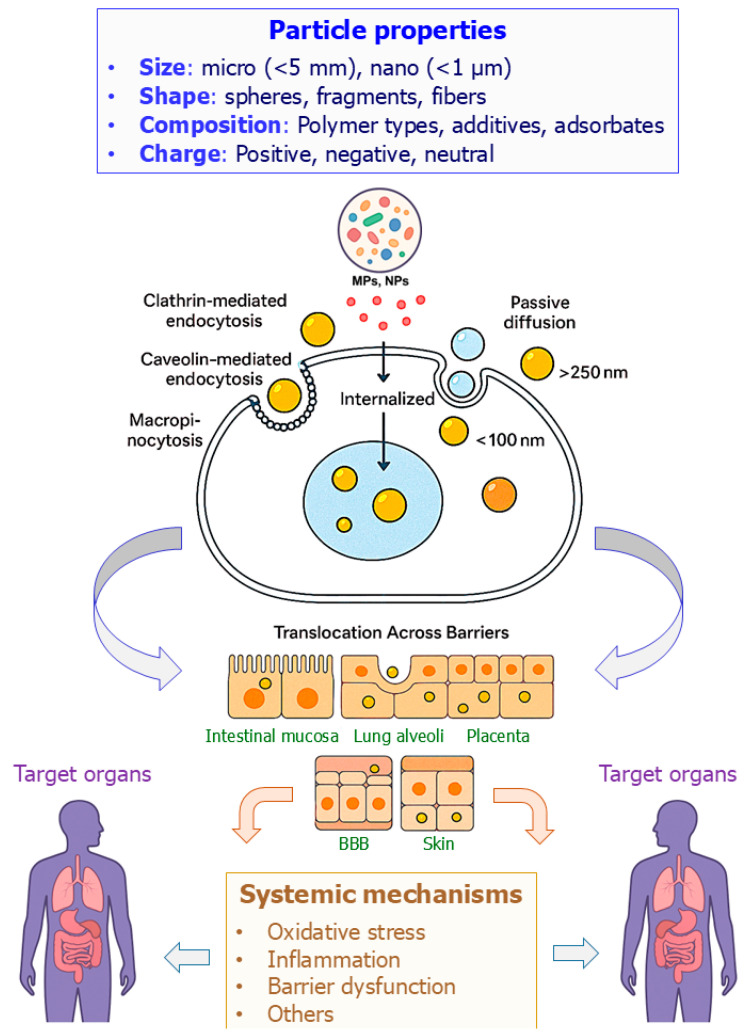
Key properties, uptake routes, and systemic mechanisms of micro- and nanoplastics (MNPs) in humans. Particle size, shape, composition, and surface properties influence uptake via ingestion, inhalation, placental transfer, and, to a lesser extent, dermal absorption. Once internalized, MNPs may trigger oxidative stress, inflammatory signaling, barrier disruption, and bioaccumulation, with downstream effects on multiple organ systems. Adapted from [20,25]. BBB: blood–brain barrier.

Once internalized, MNPs can translocate across multiple epithelial and endothelial barriers, such as the intestinal mucosa, pulmonary alveoli, placental interface, blood–brain barrier (BBB), and even dermal layers like skin [22,23]. In skin models lacking the stratum corneum, PS-NPs have been shown to penetrate the tissue [24] (Figure 1).

Mechanistic studies further demonstrated that PS-NPs in the 50–100 nm range can traverse gut and lung epithelial models, as well as cross the placental and BBB in both in vitro and in vivo systems. In human cerebral microvascular endothelial cells (hCMEC/D3), 50 nm PS-NPs induce ROS, activate NF-κB signaling, disrupt tight junction proteins, and promote necroptosis. Surface modifications, such as amine (PS-NH_2_) or carboxyl (PS-COOH) groups, exacerbate these effects [11,26,27].

## 4. Systemic (Non-Organ-Specific) Effects

Non-organ-specific effects refer to cellular and molecular mechanisms that operate broadly across various tissues, contributing to systemic pathology throughout the body. These mechanisms, detailed in Table 1, and below, can lead to systemic dysfunction regardless of the primary organ involved.

### 4.1. Oxidative Stress and Mitochondrial Dysfunction

MNPs trigger oxidative stress primarily through mitochondrial impairment. Once internalized, they localize to mitochondria and disrupt the electron transport chain (ETC), particularly complexes I and III, leading to elevated ROS production [28]. This includes superoxide anions (O_2_^−^), hydrogen peroxide (H_2_O_2_), and hydroxyl radicals (^•^OH), which overwhelm the cellular antioxidant defenses such as superoxide dismutase (SOD), catalase (CAT), and glutathione peroxidase (GPx) [29]. The resulting redox imbalance promotes oxidative damage to lipids (lipid peroxidation), proteins, and DNA, hallmarks of MNPs-induced cytotoxicity.

In HepG2 cells, exposure to 21 nm PS-NPs induced mitochondrial membrane depolarization, ATP depletion, cytochrome c release, and caspase-9 and -3 activation [30]. MNPs also disturb mitochondrial dynamics by upregulating dynamin-related protein 1 (Drp1) and downregulating mitofusin-2 (Mfn2) and optic atrophy 1 (OPA1), which are essential for outer and inner mitochondrial fusion, respectively [50]. This imbalance shifts the dynamic equilibrium toward excessive mitochondrial fragmentation, compromising cristae integrity, reducing bioenergetic efficiency, and increasing susceptibility to apoptosis.

Additional consequences include increased plasma membrane permeability, calcium imbalance, endoplasmic reticulum (ER) stress, and genotoxicity. These responses differ by cell type, plastic size, surface chemistry, and exposure duration. For instance, Caco-2 cells display altered mitochondrial potential without significant ROS elevation [51]. Environmental aging of plastics enhances surface reactivity and ROS production [29].

### 4.2. Inflammatory Responses and Immune Activation

#### 4.2.1. Inflammatory Responses

MNPs consistently induce inflammatory responses across diverse biological systems, primarily mediated through oxidative stress and pattern recognition receptors (PRRs) activation, which play an essential role in the early phase of innate immune defense. Following cellular uptake via endocytosis or phagocytosis, MNPs can disrupt membrane integrity and induce mitochondrial and lysosomal dysfunction, which activate inflammation-related signaling pathways, including NF-κB and the NLRP3 inflammasome [31,32].

**Figure 2:**
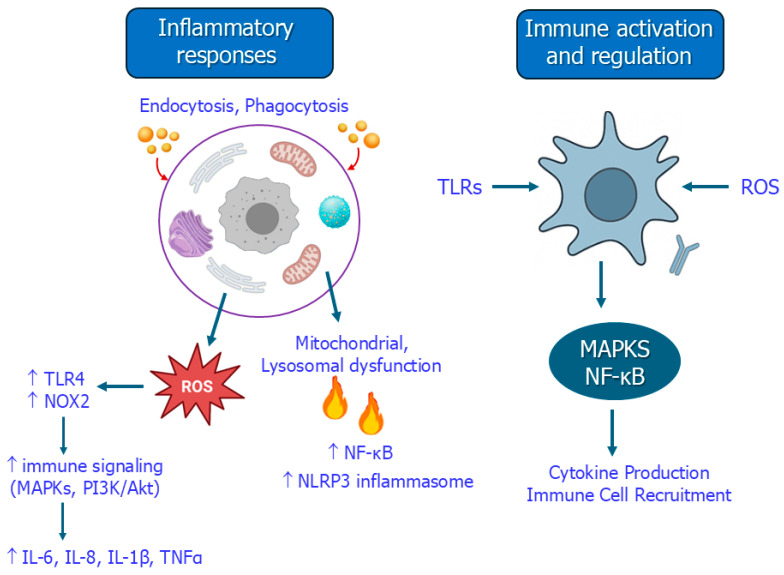
Mechanisms by which MNPs trigger inflammatory responses and immune activation. TLRs: toll-like receptors; NOX2: NADPH oxidase; MAPKs: mitogen-activated protein kinase; NF-κB: nuclear factor kappa-light-chain-enhancer of activated B cells; NLRP3: NOD-, LRR- and pyrin domain-containing protein 3; IL-6: interleukin 6; IL-8: Interleukin-8; IL-1β: Interleukin-1 beta; TNF-α: tumor necrosis factor alpha.

ROS-dependent activation of innate immune sensors, such as toll-like receptor 4 (TLR4) and NADPH oxidase (NOX2), initiates immune signaling through MAPKs, and PI3K/Akt pathways and induces the release of proinflammatory cytokines (IL-6, IL-8, IL-1β, TNF-α) [52] (Figure 2). In human-relevant models, such as HepG2 cells and endothelial cells, PS-NPs have been shown to induce VEGF expression and cytokine release, contributing to hepatic and vascular inflammation [26,30].

Particle characteristics (morphology and chemistry) influence inflammatory potential. Irregular or aged MNPs elicit greater cytokine release than pristine, spherical counterparts [53], while polyethylene terephthalate (PET)-rich particles from environmental sources evoked stronger immune responses than lab-generated plastics [54].

Inflammatory activation is also observed in reproductive and placental models. PS-MP-induced testicular premature aging has been shown to depend on the Ca^2+^/ROS/NF-κB signaling axis [55], while in placental systems PS-NPs trigger cytokine release and promote mitochondrial apoptosis, thereby contributing to infertility and pregnancy loss [56,57]. In the gastrointestinal tract, co-exposure with lipopolysaccharide (LPS) enhances inflammation via NF-κB/NLRP3 signaling and disrupts intestinal tight junctions, thus increasing intestinal permeability [31]. Persistent inflammatory responses driven by DNA damage, ER stress, and autophagy disruption contribute to cellular senescence, tissue fibrosis, and possible transgenerational effects [12].

#### 4.2.2. Immune Activation and Regulation

Beyond inflammation, MNPs exert broader immunotoxic effects by disrupting innate and adaptive responses and compromising systemic immune homeostasis. These effects are initiated through ROS generation and PRR signaling (e.g., TLRs), which in turn trigger downstream MAPKs and NF-κB signaling pathways. This cascade promotes proinflammatory cytokine production and immune cell recruitment, contributing to chronic inflammation [33,34] (Figure 2).

Particle composition and morphology markedly influence immune outcomes; irregular polyvinyl chloride (PVC) fragments and environmentally aged MNPs elicit stronger cytokine profiles in monocytes and dendritic cells than spherical PS or poly (methyl methacrylate (PMMA) particles, an effect further amplified when pollutants are adsorbed onto the particle surface [53,54].

Macrophages play a central role in mediating these effects. Particle internalization promotes M1 polarization (a proinflammatory, activated phenotype), leading to cytokine secretion, mitochondrial injury, and oxidative stress. This process drives downstream metabolic disturbances, including adipogenesis and insulin resistance, thereby linking MNP exposure to immunotoxicity and cardiometabolic risk [58]. At the molecular level, these effects are compounded by direct physicochemical interactions of MNPs with biological membranes and proteins, which alter membrane fluidity, destabilize protein structure via corona formation, and amplify oxidative and inflammatory signaling [25].

Systemic immunomodulation has been observed in animal models, including leukopenia, lymphocyte depletion, neutrophilia, and altered hematopoiesis [34]. In aquatic vertebrate models, MNP exposure modifies phagocytosis, increases IgM expression, and disrupts T-cell receptor activation, indicating impacts on both innate and adaptive immune modulation [59].

At the organ level, MNPs-induced immune responses manifest in multiple systems. Tissue-specific immune responses include Sertoli cell inflammation in testes via the Ca^2+^/mtROS/NF-κB axis [55], endothelial pyroptosis in cardiovascular tissues [60], and neuroimmune activation in the central nervous system, where plastic particles have been detected in brain tissue alongside elevated inflammatory markers in patients with neurodegenerative disease [61].

The mechanisms through which MNPs induce inflammatory responses and immune activation are summarized in Figure 2.

### 4.3. Genotoxicity

MNPs induce genotoxic effects through a network of interlinked mechanisms including oxidative DNA damage, mitochondrial dysfunction, and inflammatory signaling. Internalized particles accumulate in mitochondria and lysosomes, leading to ROS overproduction eventually leading to oxidative lesions, such as single- and double-strand DNA breaks, and micronucleus formation [34,35]. DNA damage markers such as γ-H2AX and micronucleus frequency are elevated in exposed cells [62,63].

Mitochondrial stress activates the cGAS–STING pathway by releasing mtDNA into the cytosol, where it is recognized as a damage signal. This detection triggers proinflammatory cytokine production, linking mitochondrial dysfunction to genotoxic stress and inflammation [36]. Suppression of DNA repair genes such as OGG1, XRCC1, PARP1, and LIG1/3 contributes to persistent lesions and cell cycle arrest [37].

Immune cells show elevated vulnerability to genotoxicity, with up to nine-fold greater risk than epithelial cells [64]. Co-exposure with pollutants such as tetrabromobisphenol A (TBBPA) exacerbate DNA damage in immune and reproductive tissues [63]. However, low-dose or cell-type-specific resistance is observed in models such as Caco-2 cells [51].

### 4.4. Endocrine Disruption

MNPs act as endocrine disruptors through direct receptor interference and as vectors for exogenous endocrine-disrupting chemicals (EDCs) like BPA, bisphenol S (BPS) and phthalates [38]. Their hydrophobic and porous surfaces allow for the accumulation and bioavailability of these persistent pollutants, which interfere with estrogen, androgen, and thyroid hormone signaling. In rodents and zebrafish, MNPs alter the hypothalamic-pituitary-gonadal (HPG) axis, suppress sex hormones, and disrupt steroidogenic gene expression [39,65].

Thyroid hormone synthesis and signaling are also disrupted, with changes in T3/T4 levels and expression of deiodinases and thyroid receptors [40,66]. Oxidative stress contributes to endocrine dysfunction by damaging endocrine tissues and impairing mitochondrial function, thereby promoting apoptosis in endocrine organs and disrupting hormone biosynthesis [67].

Epigenetic modifications, including DNA methylation and histone changes in gonadal tissues, have been reported in NP-exposed animals [68], with implications for transgenerational effects. MNPs can cross the BBB and influence neuroendocrine signaling, including gonadotropin-releasing hormone (GnRH) regulation [19]. Detection of MNPs in placenta, breast milk, and fetal tissue raises concern for perinatal hormonal disruption and potential long-term developmental impacts [69].

### 4.5. Autophagy, Apoptosis, and Other Regulated Cell Death Pathways

#### 4.5.1. Apoptosis

MNPs may initiate intrinsic apoptotic pathways via mitochondrial membrane depolarization, ATP depletion, and cytochrome c release, followed by caspase-9 and -3 activation. ROS production and mitochondrial fission (via Drp1) are critical mediators [30]. These effects extend to neuronal [11], placental [57], ovarian [70], intestinal [41], and renal cells [71].

#### 4.5.2. Autophagy Dysregulation

MNPs upregulate autophagy markers (LC3B, Beclin1) but impair autophagic flux, leading to p62 accumulation and reduced lysosomal clearance [42]. This autophagy disruption, mediated in part by inhibition of the PI3K/Akt/mTOR axis, further exacerbates inflammatory cytotoxicity in macrophage models [43].

Other forms of programmed cell death include ferroptosis [44], necroptosis [72], and pyroptosis [73], depending on exposure intensity and cellular context.

#### 4.5.3. Ferroptosis via Iron Dysregulation and Lipid Peroxidation Disruption

PS-MNPs have been shown to induce ferroptosis in reproductive cells by disrupting mitochondrial function, increasing ROS and lipid peroxidation, and altering iron homeostasis. In porcine oocytes, PS-NPs impaired mitochondrial membrane potential and triggered ferroptosis through elevated expression of transferrin receptor (TfRC), SLC7A11, and ACSL4, ultimately compromising oocyte maturation. Notably, these effects were significantly reversed by ferrostatin-1 (Fer-1), a ferroptosis inhibitor [44]. Similarly, in murine granulosa cells co-exposed to polystyrene microparticles (PS-MPs) and di (2-ethylhexyl) phthalate (DEHP), ferroptosis was characterized by iron overload, ROS generation, mitochondrial damage, and CYP2E1 upregulation. Fer-1 treatment partially rescued meiotic arrest and reduced necroptosis in this model [72]. Furthermore, in mice, combined exposure to MNPs and endocrine disruptors has been associated with disrupted spermatogenesis, suggesting that ferroptotic mechanisms may also impair male reproductive function [45].

Ferritinophagy via NCOA4 increases labile iron, amplifying Fenton-driven lipid peroxidation [46]. ER stress and glutathione depletion contribute to ferroptotic execution [74]. Ferroptosis is also observed in pulmonary [75] and hepatic models, with protective effects from deferoxamine and NAC [76].

In neuronal cell models (SH-SY5Y neurons and BV2 microglia), PS-NPs elicited neuroinflammatory responses and multiple forms of cell death responses, including mitochondrial dysfunction, ferroptosis, apoptosis, and pyroptosis. These effects are mediated by PERK–NF-κB activation, lipid peroxidation, and Drp1-mediated mitochondrial fission, a process that segregates damaged mitochondrial fragments for clearance via mitophagy. Pyroptosis, in particular, represents an intensely pro-inflammatory form of programmed cell death, further amplifying neuroimmune activation [77,78,79].

### 4.6. Microbiome Disruption

Emerging evidence demonstrates that micro- and nanoplastics (MNPs) disrupt host–microbiota homeostasis through interconnected mechanisms, including direct microbial interference, compromised intestinal barrier function, immune activation, and disrupted microbial metabolite production.

MNPs exert size- and charge-dependent toxicity, with smaller or positively charged particles (e.g., PS-NH_2_, ~70 nm) causing greater harm [47]. They disrupt gut microbiota by decreasing beneficial short-chain fatty acids (SCFA)-producing bacteria (e.g., Lactobacillus, Ruminococcus) while increasing pro-inflammatory taxa such as Escherichia-Shigella [48,80]. This dysbiosis compromises intestinal barrier integrity (manifested by reduced mucus secretion, tight junction breakdown, and increased permeability) leading to endotoxemia and systemic inflammation [47,81]. Additionally, such functional impairments affect host energy balance, immune tolerance, and neurotransmitter biosynthesis, contributing to widespread metabolic and neuroimmune disturbances [82,83,84]. The causal role of dysbiosis has been confirmed by fecal microbiota transplantation from MNPs-exposed mice into germ-free recipients, which replicates metabolic disruptions including weight gain and lipid dysregulation [85].

MNP-induced dysbiosis also impacts the gut–brain axis. Depletion of SCFA-producing bacteria and expansion of pathogenic taxa alter microbial neuroactive metabolite profiles. These alterations are associated with behavioral deficits, neuroinflammation, and changes in gene expression in brain regions linked to cognition and mood regulation [49,86].

In addition to these conventional mechanistic insights, recent advances in omics technologies have begun to reveal systems-level molecular perturbations associated with MNPs exposure, providing a complementary perspective on toxicity mechanisms and potential biomarkers.

### 4.7. Omics Insights into MNPs Toxicity

In recent years, omics approaches have expanded understanding of MNPs’ toxicity beyond classical endpoints. Transcriptomic profiling in cell and animal models has revealed altered expression of genes involved in oxidative stress, immune activation, lipid metabolism, apoptosis, and DNA repair following MNPs exposure [87,88]. For example, RNA-seq analyses identified deregulation of ferroptosis and p53 signaling pathways in intestinal epithelia, while small RNA profiling highlighted miRNA-mediated modulation of vascular inflammation [89]. Proteomics has provided complementary insights, revealing perturbations in mitochondrial energy metabolism, cytoskeletal remodeling, and endoplasmic reticulum stress pathways [90]. Metabolomic analyses further demonstrate disruption of amino acid, lipid, and redox metabolism, consistent with impaired glutathione homeostasis and increased oxidative burden [35,91]. Multi-omics integration is beginning to emerge, offering a more holistic perspective on how plastics perturb biological systems across multiple layers of regulation [92]. Taken together, these studies provide systems-level evidence that complements conventional toxicology, highlights novel candidate pathways for mechanistic exploration, and points toward potential molecular biomarkers that could inform future human biomonitoring and risk assessment.

Overall, studies consistently implicate oxidative stress, inflammatory signaling, and barrier dysfunction as key mechanisms of MNPs toxicity. However, comparability across studies is limited by heterogeneous particle types, concentrations and exposure durations. Many mechanistic endpoints have been observed under acute or high-dose conditions, while environmentally relevant chronic exposures are less frequently tested. For humans, current evidence indicates systemic presence (e.g., blood, placenta) [2,3], but causal links to disease endpoints remain preliminary. These findings highlight oxidative stress and inflammation as the most plausible cross-cutting pathways, while emphasizing the need for standardized exposure metrics to clarify clinical relevance.

Representative in vitro studies detailing experimental models, particle characteristics, concentrations, assessed endpoints, and key findings are summarized in Table 2.

## 5. Organ-Specific Effects

MNPs pose emerging risks to human health by affecting both specific organs and systemic biological pathways. These effects are mediated by common mechanisms described earlier, including oxidative stress, mitochondrial dysfunction, inflammation, endocrine disruption, genotoxicity, and cell death pathways, including apoptosis, autophagy disruption, and ferroptosis. Many of these mechanisms act across organ systems, reinforcing the idea that MNPs’ toxicity is not confined to isolated anatomical sites but is inherently multisystemic. The main organs/systems alongside the underlying mechanisms and functional outcomes are detailed in Table 3.

### 5.1. Gastrointestinal System

The gastrointestinal (GI) tract is a primary site of MNP entry, particularly through ingestion. Once in the lumen, MNPs can accumulate within the intestinal mucosa, where they compromise epithelial barrier integrity and increase intestinal permeability. Mechanistically, these effects are driven by oxidative stress and mitochondrial dysfunction, leading to excessive ROS generation, ATP depletion, ER stress, and disruption of tight junction proteins [31,92]. These cellular disturbances activate pro-inflammatory signaling pathways, including NF-κB and the NLRP3 inflammasome, resulting in increased secretion of cytokines such as IFN-γ, IL-6, and TNF-α [31].

In parallel, MNPs disrupt gut microbial composition, promoting dysbiosis and exacerbating barrier dysfunction. Dysbiosis typically involves a reduction in beneficial taxa and an enrichment of pro-inflammatory species, leading to impaired production of key microbial metabolites, particularly SCFAs, and contributing to mucosal immune imbalance and chronic low-grade inflammation [105,106,107]. These microbiota-mediated effects are increasingly recognized as systemic, influencing not only GI health but also metabolic, neurobehavioral, and reproductive functions through the gut–brain and gut–gonadal axes [32,59].

The convergence of oxidative stress, ER and mitochondrial dysfunction, barrier compromise, cytokine release, and particle translocation underpins MNP-induced gastrointestinal toxicity. These mechanisms have been consistently demonstrated in animal models and validated using advanced human-derived experimental systems [97]. However, human data remain limited, and the long-term effects of chronic, low-dose MNPs exposure are still poorly characterized [108].

New mechanistic studies have shown a clear size-dependence of intestinal toxicity: nanosized plastics (~100 nm) induce ferroptosis via p53–Fosl1 pathways, while larger microsized particles (~10 µm) cause epithelial disruption and metabolic reprogramming through YAP activation [109]. These findings underscore that particle size critically influences mode of injury.

### 5.2. Respiratory System

Inhaled MNPs, particularly PS-NPs, pose a growing respiratory health concern in urban and occupational settings due to their deposition in the respiratory tract. Once deposited, these particles disrupt epithelial barrier integrity and trigger local inflammation through mechanisms comparable to those observed in the gastrointestinal system. Key pathways include mitochondrial dysfunction, excessive ROS production, disrupted calcium homeostasis, and ER stress [98].

In human airway epithelial co-culture models, PS-NPs impaired autophagic flux and induced ER stress, resulting in upregulation of IL-33 and release of alarmins, which activate innate immune signaling and promote epithelial hyperreactivity [110]. In addition, inhaled MNPs activate the NLRP3 inflammasome, amplifying the secretion of pro-inflammatory cytokines and contributing to subacute airway inflammation [110].

Recent studies also implicate ferroptosis, a form of iron-dependent cell death driven by lipid peroxidation and suppression of glutathione peroxidase 4 (GPX4), as a key mechanism of MNP-induced pulmonary toxicity [75]. These cellular stress pathways collectively lead to immune cell infiltration, alveolar epithelial damage, and fibrotic remodeling, ultimately impairing gas exchange efficiency.

Chronic exposure to airborne MNPs has been associated with the development or exacerbation of respiratory diseases, including asthma and chronic obstructive pulmonary disease (COPD), and may contribute to carcinogenesis through persistent inflammation and epithelial injury [111]. Furthermore, inhaled nanoplastics have been shown to translocate across the alveolar–capillary barrier, suggesting the potential for systemic distribution and extrapulmonary effects [99].

### 5.3. Cardiovascular System

MNPs, particularly PS-NPs, can translocate into the bloodstream and accumulate in cardiac and vascular tissues, where they initiate endothelial injury and promote vascular inflammation. Experimental evidence from both in vitro and in vivo models implicates MNPs in cardiovascular toxicity, demonstrating that their accumulation triggers a cascade of molecular and cellular disturbances. These include oxidative stress, mitochondrial dysfunction, calcium homeostasis disruption, and ER stress. These early perturbations activate inflammatory signaling via NF-κB and TGF-β1/Smad, and activate Wnt/β-catenin signaling, all of which contribute to myocardial fibrosis and vascular pathology [45,93,100,101,112].

In vitro models, including cardiac organoids and human iPSC-derived cardiomyocytes, show that even low-dose PS-NP exposure disrupts contractility, impairs calcium handling, and elevates mitochondrial ROS levels. These changes lead to reduced membrane potential, energy failure, and upregulation of hypertrophic and stress markers like pro–B-type natriuretic peptide (proBNP), indicating a decline in myocardial function [93,113]. In vivo studies in ApoE^−^/^−^ mice show that chronic oral PS-NP exposure accelerates atherosclerotic plaque formation via increased smooth muscle cell migration regulated by kinesin family member 15 (KIF15) [101].

In vascular endothelium, exposure to different types of NPs leads to oxidative stress, impaired cell migration, and inflammatory signaling mediated by CEBPB–miR-1908-5p interactions, supporting endothelial dysfunction as a plausible mechanism of cardiovascular risk [89].

Additionally, MNPs impair autophagic flux, promote apoptosis, and activate stress-response pathways such as TNF-α/NF-κB and P38/MAPK, which may drive inflammation, arrhythmogenic remodeling, and cardiac fibrosis [114,115]. Rodent studies also reveal mitochondrial swelling, fibrosis, and dysregulated expression of apoptosis-related genes [116]. Although experimental studies indicate mechanisms (oxidative stress, endothelial injury, fibrosis) that could contribute to arrhythmogenic remodeling and atherogenesis, direct causal human evidence remains limited and predominantly correlative.

Overall, these findings support a multi-step model of MNPs-induced cardiotoxicity in which particle accumulation leads to organelle dysfunction, which drives oxidative and ER stress. These in turn activate inflammatory and fibrotic pathways, ultimately resulting in structural and functional cardiac impairment.

### 5.4. Nervous System

MNPs are increasingly recognized for their neurotoxic potential due to their ability to cross biological barriers, accumulate in regions such as the hippocampus and cortex, and impair central nervous system function. These particles induce neurotoxicity through multiple interconnected mechanisms including oxidative injury, mitochondrial dysfunction, neuroinflammation, and disrupted gene expression [11]. MNPs generate excessive ROS, leading to mitochondrial dysfunction, lipid peroxidation, and DNA damage [102,117]. In murine models, PS-NPs have been shown to disrupt spatial memory and synaptic integrity via mitochondrial injury and ROS production [118]. Similarly, zebrafish models revealed reduced locomotor activity, apoptosis, and altered neurotrophic signaling, including decreased brain-derived neurotrophic factor (BDNF) [119].

In vitro studies using human SH-SY5Y neurons further demonstrate activation of AMPK/ULK1-mediated mitophagy, resulting in mitochondrial fragmentation and neuronal apoptosis [120]. Disruption of autophagic balance may contribute to neurodegeneration-like phenotypes. Neuroinflammation also plays a central role, with microglial activation and NF-κB signaling implicated in elevated pro-inflammatory cytokines and depressive-like behaviors in mice [11,121].

Developmental exposure to MNPs disrupts neurotransmitter systems, including GABA and acetylcholine, leading to behavioral abnormalities like anxiety, depression-like behavior, and impaired social interactions in offspring [122]. Exposure to polypropylene (PP)-NPs in both mouse models and human brain organoids further impairs neurodevelopment by inhibiting neuronal differentiation, reducing neural stem cell proliferation, and impairing cognitive and motor functions. These effects are mechanistically linked to dysregulation of neurodevelopmental signaling pathways, and significant downregulation of neuronal markers of neuronal differentiation and maturation, including TUJ1, MAP2, and PAX6. In addition, molecular analyses identified CYSLTR1 and PTH1R as probable molecular targets of PP-NPs, suggesting receptor-mediated pathways as key contributors to the observed neurotoxicity [94].

Together, these findings underscore the vulnerability of the developing brain to NPs exposure and highlight the need for preventive strategies to safeguard fetal neurodevelopment during pregnancy.

### 5.5. Reproductive System

MNPs pose significant risks to reproductive health by affecting gametogenesis, hormone production, placental function, embryogenesis and fetal development. The primary upstream mechanisms, oxidative stress and endocrine disruption, initiate a cascade of cellular dysfunctions in reproductive tissues [103].

MNPs adversely affect both male and female reproductive systems through a convergence of oxidative, inflammatory, and epigenetic mechanisms. In females, MNPs exposure interferes with folliculogenesis and oocyte maturation, primarily through oxidative stress, mitochondrial dysfunction, and ferroptosis [44]. In placental tissues, MNPs induce autophagy inhibition, inflammasome activation, and necroptosis, leading to impaired hormone synthesis, disrupted cytokine signaling, reduced nutrient transport, and fetal growth restriction [95].

In males, MNPs compromise the blood–testis barrier and damage Sertoli and Leydig cells, resulting in decreased testosterone synthesis and impaired sperm quality. Mechanistically, these effects are mediated by mitochondrial and lysosomal damage, ROS overproduction, MAPK-Nrf2 pathway activation, and apoptotic signaling [55,104]. Lipid peroxidation further exacerbates cellular injury and hormonal imbalance.

Beyond direct organ toxicity, MPs can translocate from parents to offspring, impairing the neurological, immune, metabolic, and reproductive systems. This raises serious concerns about transgenerational toxicity. While in vitro and organoid models have shown MP-induced harm in human-derived germ cells and placental tissues, evidence on long-term and heritable effects remain insufficiently characterized [123].

Collectively, these findings highlight that MNP-induced reproductive toxicity arises from ROS-driven cell death pathways, endocrine disruption, and heritable epigenetic modifications, affecting fertility and posing potential transgenerational risks in both sexes.

Overall, organ-focused studies highlight several particularly vulnerable systems, including the gastrointestinal tract, liver, reproductive and endocrine organs, placenta, cardiovascular system, and central nervous system. Across these targets, recurring findings involve barrier disruption (intestinal, vascular, placental), oxidative and inflammatory injury in hepatic and cardiac tissues, reproductive dysfunction, and neurobehavioral alterations. However, mechanistic and clinical correlations remain scarce, and causal links to specific disease outcomes are not yet established. Translational interpretation must therefore remain cautious: while animal and in vitro models provide strong mechanistic plausibility, definitive connections to human disease will require longitudinal biomonitoring and well-designed epidemiological studies.

A structured overview of available human biomonitoring studies is presented in Table 4. These studies confirm the presence of MNPs in multiple human matrices, including lungs, blood, placenta, feces, liver, kidney, and brain. Common polymers such as polyethylene, polypropylene, and PET indicate widespread exposure via inhalation and ingestion. While diverse analytical methods strengthen confidence in detection, studies remain small, methodologically variable, and largely descriptive. Evidence of MNPs in sensitive tissues suggests potential health relevance, emphasizing the need for standardized methods, larger cohorts, and integrated mechanistic studies to assess human health impacts.

Complementary findings from in vivo animal models, including exposure routes, dose ranges, affected organs, and mechanistic outcomes, are summarized in Table 5.

**Figure 3:**
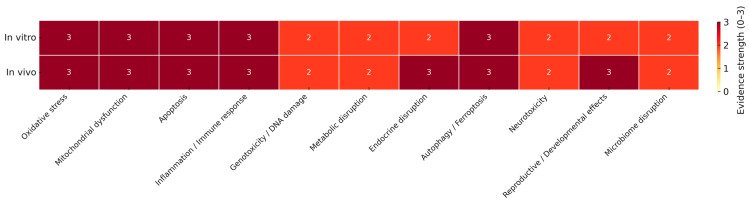
Composite heatmap of adverse effects reported in in vitro and in vivo studies of micro- and nanoplastics (MNPs). It summarizes the frequency and consistency of major toxicological endpoints across these evidence streams. Endpoints include oxidative stress/ROS production, mitochondrial dysfunction, apoptosis, inflammation/immune activation, genotoxicity/DNA damage, metabolic disruption, endocrine disruption, autophagy/ferroptosis, neurotoxicity/behavioral effects, reproductive and developmental effects, and microbiome disruption. Color scale: 0 = not reported, 1 = occasional evidence, 2 = moderate evidence, 3 = consistent evidence.

To provide an integrated overview of the concordance between in vitro and in vivo findings, a composite heatmap was developed to summarize the frequency of major toxicological endpoints (Figure 3).

Taken together, evidence across study types highlights converging mechanisms of MNPs toxicity across experimental models. Human biomonitoring (Table 4) and human-derived systems (Table 2) demonstrate the presence of particles in key biological matrices and provide early indications of associated inflammatory and functional disturbances. Mechanistic studies consistently implicate oxidative stress, mitochondrial dysfunction, apoptosis, ferroptosis, and immune activation as central pathways, which are summarized in Table 1. In vivo studies (Table 5) extend these findings, documenting systemic effects on reproductive, endocrine, cardiovascular, and neurological systems, as well as microbiome-mediated disturbances (Table 3). To illustrate the relative consistency of these effects across evidence streams, a composite heatmap of major endpoints is shown in Figure 3, highlighting oxidative stress, mitochondrial dysfunction, inflammation, and cell death as the most robustly supported toxicological pathways. Collectively, these convergent findings strengthen the mechanistic plausibility of adverse health effects from MNPs exposure while identifying critical knowledge gaps that warrant further investigation in human populations.

## 6. Challenges and Methodological Limitations

### 6.1. Standardization and Reporting Challenges

Research on MNPs is limited by the lack of standardized definitions and comprehensive physicochemical characterization of particles, including size, shape, polymer type, surface chemistry, and bio-corona composition. This heterogeneity contributes to substantial inter-laboratory variability and reduces reproducibility. NPs, in particular, exhibit diverse morphologies and surface properties, such as charge and hydrophobicity, which critically influence their environmental behavior and biological interactions [124,125,126,127].

A further limitation is the absence of harmonized protocols for particle characterization and exposure reporting. Many studies use inconsistent exposure metrics (e.g., mass, particle number, surface area), and particle characterization is often incomplete, missing key parameters such as size distribution and surface chemistry. In animal studies, additional methodological shortcomings include small sample sizes, lack of randomization and blinding, and the use of high acute doses without internal dose measurements. These issues constrain causal inference, reduce reproducibility, and complicate cross-study comparisons.

To address these challenges, the adoption of harmonized reporting frameworks, such as the OHAT (Office of Health Assessment and Translation) and SYRCLE (Systematic Review Centre for Laboratory Animal Experimentation), is essential. Furthermore, prioritizing studies with thorough particle characterization and environmentally relevant exposures will improve the reliability, reproducibility, and comparability of MNPs research findings.

### 6.2. Exposure Assessment and Detection Limitations

Exposure assessment is complicated by wide variations in experimental design. Many in vitro studies use unrealistically high doses that poorly reflect environmentally or physiologically relevant scenarios, limiting the extrapolation of results to human health. The scarcity of robust in vivo human studies further constrains understanding of systemic and long-term effects.

Detection and quantification of NPs in biological tissues and environmental matrices remains technically challenging due to their minute size and the complexity of sample composition [128,129]. Current analytical methods [130] often lack the sensitivity and specificity to reliably measure internal doses, biodistribution, or the potential for bioaccumulation and biomagnification. These analytical gaps impede accurate exposure assessment and progress toward realistic dose–response relationships.

### 6.3. Mechanistic Understanding and Research Priorities

Biological interaction studies remain limited by incomplete mechanistic understanding of how nanoplastics enter cells, cross biological barriers, and disrupt molecular pathways. The lack of harmonized endpoints for NPs’ toxicity (e.g., standardized measures of uptake, oxidative stress, inflammation, genotoxicity, apoptosis) further complicates data synthesis and cross-study comparison [96].

Addressing these challenges will require more physiologically relevant experimental systems, improved detection platforms, and harmonized mechanistic endpoints. Examples of emerging solutions are discussed in Section 7.

### 6.4. Relevance of Experimental Doses to Human Exposure

A major challenge in interpreting the toxicological evidence on MNPs is the disconnect between the high, often acute, concentrations used in experimental models and the lower, chronic exposures experienced by humans in real-world settings. Recent assessments estimate that adults may ingest on the order of 10^3^–10^5^ particles per day (equivalent to μg–mg/day depending on particle size and density) and inhale a similar magnitude annually [131,132,133]. By contrast, many mechanistic studies have employed “pristine” spherical polystyrene particles at much higher concentrations (e.g., 10^6^–10^9^ particles/mL in vitro; >10^6^–10^8^ particles/kg/day in vivo), primarily to elucidate molecular pathways. While informative, such high-dose designs often exceed environmentally relevant exposures.

To provide a clearer comparison, Table 6 summarizes estimated human exposure ranges via ingestion, inhalation, and dermal routes alongside typical experimental doses. These comparisons highlight that experimental dosing is frequently several orders of magnitude higher than environmental estimates, especially for ingestion and inhalation. In contrast, dermal exposure under environmental conditions remains poorly characterized, and available studies often rely on high-dose topical or subcutaneous administration, limiting translational relevance.

Many studies on MNPs use exposure doses that exceed estimated environmental concentrations. Relatively few investigations have examined chronic, low-dose exposures that more closely reflect real-world conditions. Although scarce, these studies are particularly valuable for risk assessment. Interpretation is further complicated by inconsistent reporting of exposure metrics (e.g., particle number, mass, and size distribution) and the lack of internal dose data, such as tissue burdens. As a result, harmonization across studies is challenging, and broad extrapolations from laboratory models to humans remain premature.

To enhance translational value, future studies should report exposures in standardized units, incorporate chronic and low-dose designs, and measure internal particle burdens whenever feasible. Such improvements are essential for aligning experimental models with human exposures and supporting more accurate health risk assessments.

### 6.5. Criteria to Distinguish Polymer-Intrinsic vs. Additive/Adsorbate Effects

Differentiating whether observed effects are driven by the polymer backbone or by associated additives and adsorbed contaminants remains a major challenge in MNPs toxicology. Rigorous attribution requires experimental designs that combine well-characterized particles (pristine and environmentally aged), detailed chemical analysis of leachates, and appropriate additive-only or vehicle controls. Endocrine disruption, oxidative stress, and genotoxic responses have in several cases been linked more convincingly to plasticizers, stabilizers, or adsorbed pollutants than to the polymer matrix. However, in the absence of such controls, attribution remains uncertain, underscoring the need for systematic leachate testing in MNP studies [135].

### 6.6. Methodological Overview of Analytical Detection Methods

Current detection approaches for MNPs in human and experimental matrices rely primarily on vibrational spectroscopy and mass spectrometry-based techniques. Fourier-transform infrared (FTIR) and Raman microspectroscopy are widely used for polymer identification but are typically limited in resolution to particle sizes > 1–10 µm (FTIR) and ~1 µm (Raman), depending on instrumentation [136]. Pyrolysis–gas chromatography/mass spectrometry (Py-GC/MS) and related thermal degradation methods enable mass-based polymer identification and quantification; however, they are destructive and provide no information on particle size or morphology [137,138]. As a result, none of these techniques currently allow routine, highly specific quantification of NPs in complex human tissues.

Persistent challenges include laboratory contamination, matrix interferences, and lack of standardized reference materials. Recent methodological innovations that aim to address these limitations are reviewed in Section 7.

Taken together, these limitations (spanning particle characterization, exposure assessment, analytical detection, mechanistic attribution, and study design) highlight the need for greater standardization and innovation. Without harmonized protocols, environmentally relevant exposure models, and robust quality controls, research on MNPs may generate fragmented evidence that is difficult to translate into reliable health risk assessments. Solutions to these challenges are considered in Section 7.

## 7. Emerging Approaches and Future Directions

Recent methodological advances are beginning to address the limitations outlined in Section 6 and are reshaping the landscape of MNPs research.

On the analytical front, advanced separation and detection platforms, including asymmetric flow field-flow fractionation coupled with multi-angle light scattering (AF4-MALS), nanoscale secondary ion mass spectrometry (Nano-SIMS), and high-resolution mass spectrometry imaging (MSI), now provide improved resolution, size discrimination, and chemical specificity for NPs detection in complex matrices [139,140,141].

At the experimental level, human-relevant in vitro systems such as intestinal and placental organoids, as well as multi-organ organ-on-chip devices, provide physiologically realistic platforms to study uptake, translocation, and mechanistic toxicity. These models better recapitulate barrier tissue architecture and function, enabling more predictive toxicological assessments.

Systems-level approaches are also advancing, with integrated multi-omics workflows (transcriptomics, proteomics, metabolomics) increasingly used to map disrupted biological pathways and identify candidate biomarkers of exposure and effect [142]. Non-invasive imaging technologies, including novel optical spectroscopy and high-resolution MSI, further enhance the ability to track NPs in biological fluids and tissues.

Beyond laboratory science, longitudinal epidemiological studies incorporating biomonitoring and mechanistic endpoints will be crucial to establish causal links between internal exposure and health outcomes. International initiatives [10,132,143] are accelerating standardization through the development of certified reference materials, harmonized guidelines, inter-laboratory validation schemes, and reporting recommendations, all critical steps toward reproducible quantification and regulatory uptake.

Finally, comprehensive regulatory and policy actions are necessary to reduce MNPs emissions at source. These include limiting plastic production, improving product design to minimize leaching, strengthening packaging standards, promoting consumer behavior change, and advancing global governance frameworks that address plastic pollution systemically and sustainably [144,145,146].

## 8. Conclusions

Emerging mechanistic evidence increasingly implicates MNPs in a broad spectrum of cellular pathologies that underlie systemic and organ-specific health outcomes. Key processes include oxidative stress, inflammation, genotoxicity, endocrine disruption, autophagy dysregulation, cellular senescence, and apoptosis. Together, these interrelated pathways highlight the widespread biological impact of MNPs exposure on human health.

Despite these advances, significant methodological challenges remain. Addressing them requires rigorous standardization of physicochemical characterization and exposure protocols, development and application of human-relevant experimental platforms, and comprehensive longitudinal epidemiological studies. To deepen mechanistic understanding, integrated multi-omics approaches are crucial for identifying molecular signatures and disrupted biological pathways linked to MNPs exposure.

Ultimately, translating mechanistic insights into reliable risk assessment will require coordinated, interdisciplinary efforts that bridge scientific research with policy development. This integrated approach is essential to inform regulatory decisions, guide public health interventions, and mitigate the growing human health risks posed by the ubiquitous presence of MNPs in the environment.

## Figures and Tables

**Table 1:** Systemic (Non-organ-specific) effects of micro- and nanoplastics (MNPs).

Mechanism	Key Features	Representative References
Oxidative stress and mitochondrial dysfunction	Mitochondrial membrane depolarization, ROS overproduction, ETC complex I/III disruption, ATP depletion, mtDNA damage	[28,29,30]
Inflammation	Activation of NF-κB, NLRP3 inflammasome, cytokine release (IL-1β, IL-6, TNF-α), TLR4 signaling, MAPK/PI3K/Akt pathways.	[31,32]
Immune activation	PRR engagement (e.g., TLRs), monocyte/dendritic cell skewing, lymphocyte depletion, neutrophilia, altered hematopoiesis and immune response, persistent low-grade inflammation.	[33,34]
Genotoxicity	DNA strand breaks, 8-OHdG formation, γ-H2AX, micronuclei, mtDNA leakage, suppression of DNA repair genes (OGG1, XRCC1, PARP1)	[35,36,37]
Endocrine disruption	Hormonal imbalance (estrogen, androgen, thyroid axes), receptor interference, altered gene expression, epigenetic changes, HPG axis disruption	[38,39,40]
Apoptosis	Intrinsic mitochondrial pathway activation, cytochrome c release, caspase-9/-3 activation, Bcl-2/Bax imbalance	[30,41]
Autophagy disruption	Impaired autophagic flux, LC3B/Beclin1 upregulation, p62 accumulation, lysosomal dysfunction, mTOR inhibition	[42,43]
Ferroptosis	Iron overload, GPX4 suppression, lipid ROS, ACSL4 and TfRC upregulation, ferritinophagy (NCOA4), glutathione depletion	[44,45,46]
Microbiome disruption	Gut dysbiosis, reduced microbial diversity, loss of beneficial bacteria, increased intestinal permeability, altered microbial metabolism, gut–brain axis disruption	[47,48,49]

**Table 2:** Representative in vitro studies on micro- and nanoplastics (MNPs) conducted in mammalian cell models. Human-derived test systems are listed first, followed by animal-derived models. Studies are organized by cell type and experimental context, and include details on particle type and size, exposure conditions, assessed endpoints, key findings, and study limitations.

Model	Particle Type and Size	Detection Method	Tested Concentration	Endpoints Measured	Major Findings	Limitations	Study (Ref.)
Human neuroblastoma SH-SY5Y cells	Polystyrene NPs (PS-NPs, 50 nm)	Not reported	20, 50, 100, 200, and 500 mg/L (for 24 h)	Cell viability, LDH release, ROS, Ca^2+^, apoptosis, mitochondrial function, autophagy markers	PS-NPs induced oxidative stress, mitochondrial dysfunction, apoptosis (via caspase pathway), and autophagy activation; NAC mitigated effects	Single cell line, high concentrations, short exposure	[79]
Human hepatocellular carcinoma (HepG2) cells	PS-NPs, 21.5 ± 2.7 nm	Transmission electron microscopy (TEM)	6.25, 12.5, 25, 50 μg/mL (24 h)	Viability, ATP, mitochondrial membrane potential) (MMP), ROS, mitochondrial fission proteins, apoptosis markers	Concentration-dependent cytotoxicity; mitochondrial damage via ROS and DRP1-dependent fission; apoptosis	Tumor-derived cell line, short-term exposure	[30]
Human colon adenocarcinoma (Caco-2) cells	PS-NPs, 0.04 to 0.09 μm (fluorescent & non-fluorescent)	TEM	25, 50, 100, 125, 150, 175 and 200 μg/mL (for 24/48 h)	Cellular uptake, Cytotoxicity, ROS, genotoxicity (MN, Comet assay), DNA oxidative damage, stress-related gene expression	PS-MNPLs internalized; induced oxidative stress and genotoxicity	No concentration-response data; limited mechanistic insight	[51]
Primary human monocytes & monocyte-derived dendritic cells	PS, PMMA, PVC NPs (50, 100, 310 nm; irregular vs. spherical)	Attenuated total reflection Fourier-transform infrared spectroscopy (ATR-FTIR), py-GC–MS,	30–300 particles/cell	Cytokine release (inflammation) (IL-6, IL-10, TNF, IL-12p70, IL-23)	PVC and irregular particles caused strongest cytokine responses; shape- and polymer-dependent effects	Donor variability, limited mechanistic data	[53]
Various human cells (THP-1 cells, PBMCs -peripheral blood mononuclear cells-, whole blood, T84 intestinal epithelial cells)	PP, PE, PS, PET MNPs (commercial and environmental water sources, mixed origins)	FTIR spectroscopy, SEM (scanning electron microscopy), XRD (X-ray diffraction), confocal imaging	100 ng/mL to 1 mg/mL	IL-1β, IL-6 secretion, cell death, morphology	PET-rich MNPs caused strongest inflammation and cell death; authentic environmental particles highly reactive	Heterogeneous MNPs mixture; limited mechanistic resolution	[54]
Human ovarian granulosa-like tumor (KGN) cells	PS-NPs, fluorescent (20 nm)	TEM, SEM	100 μg/mL (for 48 h)	Proliferation, apoptosis, ROS, Hippo signaling (MST1, LATS1, YAP1)	NPs internalized; induced ROS, apoptosis, Hippo pathway dysregulation; salidroside attenuated effects	Cancer-derived line, high exposure concentration	[56]
Normal human hepatic (L02) & lung (BEAS-2B) cells	PS-NPs, 80 nm	Fluorescent labeling and TEM, metabolomics	0.006, 0.0125, 0.03125, 0.0625, 0.125, or 0.25 mg/mL (for 24 h)	Mitochondrial function, ROS, respiration, metabolomic changes	NPs internalized; mitochondrial dysfunction and oxidative stress, especially in L02 cells	Short exposure; polymer unspecified	[35]
Human peripheral lymphocytes	PS-NPs, 50 nm (45–68 nm)	TEM, SEM	0.001, 0.01, 0.1, 1, 10, 100 μg/mL (for 24–48 h)	Cytotoxicity, genotoxicity (DNA damage)	Significant DNA damage and mitotic inhibition at most concentrations	No mechanistic data beyond genotoxicity	[62]
Human iPSC-derived kidney organoids	PS-MPs, various (1 μm)	TEM, FTIR	1.25, 2.5, 5, 10, 20 μg/mL (for 48 h)	Differentiation, apoptosis, mitochondrial function, signaling pathways	PS-MPs impaired nephron development, induced mitochondrial apoptosis via Bcl-2/Bax/caspase pathway	Developmental model only; no chronic exposure	[71]
Human iPSC-derived cardiomyocytes (hiPSC-CMs)	PS MNPs (1 μm and 0.05 μm)	SCIOS Dual-Beam SEM, Raman microscopy (image and spectra)	0.1, 1, 10 and 1000 μg/L (for 7, 10 15 and 20 days)	Cell viability, contractility, Ca^2+^ transients, mitochondrial function (MMP, mitochondrial ROS)	Dose-dependent reduction in contractility, ROS increase, mitochondrial dysfunction	Only PS tested; long-term but low-dose limited	[93]
Human cerebral organoids	PP-NPs (100 nm)	TEM, Laser Granulometer (LS230)	0, 10, 25, and 50 µg/mL (for 30 days)	Growth, neuronal differentiation, gene expression	Impaired neurogenesis and differentiation; altered neuroactive ligand–receptor pathways	No particle quantification in organoids; limited dose metrics	[94]
Human placental chorionic villi explants	PS-MPs, 5 μm	Raman spectroscopy (XploRA Raman spectrometry) 1H NMR, biochemical assays	1, 10, 100 μg/mL (for 24 h)	Cytotoxicity, oxidative stress, antioxidant activity, metabolomics	Dose-dependent cytotoxicity and oxidative stress; disrupted antioxidant balance and metabolism	Ex vivo explant model; acute exposure	[95]
Human PBMCs, HMC-1, RAW 264.7 (murine)	PP microplastics, 20–200 μm	SEM, field emission scanning electron microscope (FE-SEM)	0, 500 μg/L. 10, 50, 100, 500, and 1000 μ g/mL (for RAW cells) (for 48 h)	Cytotoxicity, cytokine secretion, ROS, proliferation	Small PP particles at high doses induced cytokine/histamine release; immune activation	Large particle size; heterogeneous preparation	[96]
Porcine aortic endothelial cells (AOC)	Polystyrene NPs (PS-NPs, 100 nm)	Fluorescence co-localization	5, 25 and 75 µg/mL (for 48 h)	Cell growth, ROS, antioxidant defense, VEGF production, metabolic activity	NPs internalized by AOC; increased VEGF, ROS, and metabolic activity; disrupted redox status	Polymer type and concentration not specified; limited mechanistic insight	[26]
Rat ovarian granulosa cells (GCs) from Wistar rats	PS-MPs, 0.5 μm	SEM, FTIR	1, 5, 25 μg/mL (for 90 days)	ROS, apoptosis, anti-Müllerian hormone (AMH), fibrosis markers	PS-MPs entered GCs, caused ROS, apoptosis, fibrosis via Wnt/β-catenin pathway; NAC reversed effects	Rat origin; in vitro data linked to in vivo exposure	[70]
Murine macrophage Raw 264.7 cells	Mixed microplastics (MPs, various polymers from dust fall, from 178.54 to 726.27 μm)	Py-GC-MS, Western blot Field-emission scanning electron microscopy (FESEM)	100 μg/g of the dust fall MP (DF-O)/MP samples (for 24 h)	Cytotoxicity, autophagy, apoptosis signaling, cytokines	MPs inhibited autophagy (↓LC3B, *p*-Akt/mTOR) and induced apoptosis (↑Bax/Bcl-2, Caspase-3)	Complex real-world MP mixture; unclear concentration	[43]
Gut bacterial cultures (*E. coli*, *L. rhamnosus*, *Erysipelotrichales*) from C57BL/6 mouse fecal samples	PS-MPs, 1 μm	Not reported	0, 10, 20, 50, 100, 500 μg/mL (for 24 h)	Growth rate, metabolomics, microbiota composition	MPs reduced bacterial growth, altered metabolism (sugar/sulfur pathways)	Bacterial model; relevance to human microbiota indirect. No particle characterization	[85]

**Table 3:** Mechanisms and Functional Effects of Micro- and Nanoplastics (MNPs) by Organ/System.

Organ/System	Key Mechanisms	Functional Outcomes	Representative References
Gastrointestinal	Oxidative stress, ER stress, mitochondrial dysfunction, barrier disruption, NLRP3 inflammasome activation, microbiota dysbiosis	Intestinal permeability, endotoxemia, inflammation, altered microbial metabolism	[31,92,97]
Respiratory	Oxidative stress, ferroptosis, mitochondrial damage, ER stress, autophagy impairment, NLRP3 inflammasome activation, barrier dysfunction	Chronic inflammation, lung fibrosis, epithelial remodeling	[75,98,99]
Cardiovascular	Mitochondrial ROS, NF-κB and TGF-β1/Smad activation, ER stress, autophagy inhibition, calcium signaling disruption, endothelial injury, Wnt/β-catenin signaling	Myocardial fibrosis, arrhythmias, endothelial dysfunction, atherosclerosis	[93,100,101]
Nervous System	BBB disruption, oxidative stress, mitochondrial dysfunction, neuroinflammation (via NF-κB), microglial activation, synaptic gene downregulation, impaired neuroplasticity	Neuroinflammation, cognitive impairment, anxiety, memory loss, impaired neurodevelopment	[11,94,102]
Reproductive System	Oxidative stress, apoptosis, ferroptosis, necroptosis, hormone synthesis disruption, placental barrier dysfunction, epigenetic alteration	Infertility, placental damage, impaired sperm quality, ovarian dysfunction, gametogenesis failure, altered hormone signaling transgenerational effects	[72,95,103,104]

**Table 4:** Summary of the human biomonitoring studies evaluating micro- and nanoplastics (MNPs) exposure. The table includes the biological matrices examined, detection methods, particle types and sizes, health endpoints, major findings, and key limitations.

Biological Matrix/Model	Particle Type and Size	Detection Method	Endpoints Measured	Major Findings	Limitations	Study (Ref.)
Human lung tissue (autopsy samples)	Polymeric particles (<5.5 µm) and fibers (8.1–16.8 µm); mainly polyethylene, polypropylene	Microscopy and polymer characterization	Presence and morphology of MNPs	Microplastics (MPs) found in human lungs, indicating inhalation exposure; particle heterogeneity may relate to respiratory effects	Small sample size; no health outcome data; contamination control challenges	[1]
Human whole blood (22 volunteers)	≥700 nm; polyethylene terephthalate, polyethylene, styrene polymers, PMMA	Double-shot pyrolysis-GC/MS	Quantification of polymeric particles	First evidence of plastic particles in human blood; suggests systemic uptake	Small sample; no temporal exposure data; unknown biological fate	[2]
Human placenta (6 donors)	5–10 µm; polypropylene and pigmented microplastics	Raman microspectroscopy	Presence, morphology, chemical composition	MPs found in maternal, fetal, and membrane sides of placenta; evidence of translocation potential	Limited sample size; contamination risk; no exposure correlation	[3]
Human feces (8 volunteers, Europe & Asia)	50–500 µm; 9 polymer types including polypropylene, PET	FTIR microspectroscopy	Presence and abundance of MPs	All samples positive for MPs; ingestion likely from food, water, and air	Very small cohort; single sample per person; no link to exposure route	[4]
Human kidney, liver, and brain (postmortem tissues)	Polyethylene-dominant MNPs, nanoscale shard-like fragments	Pyrolysis-GC/MS, ATR-FTIR, electron microscopy with EDS	Tissue concentrations, morphology, composition	Confirmed MNP presence in deep tissues; higher polyethylene in brain; possible link with dementia	Cross-sectional design; postmortem contamination control; mechanistic uncertainty	[46]

**Table 5:** Summary of the representative in vivo studies on micro- and nanoplastics (MNPs). This table compiles key animal studies investigating the systemic and organ-specific toxicity of MNPs. It includes details on the study model, particle type and size, route and duration of exposure, assessed endpoints, major mechanistic findings, and identified limitations.

Species/Model	Particle Type and Size	Dose and Route	Duration	Organ/System Studied	Endpoints Measured	Major Findings	Limitations	Study (Ref.)
Mouse (C57BL/6)	Polystyrene nanoplastics (PS-NPs), 100 nm	Intraperitoneal injection (5 μg/g); with/without LPS	Every other day for 2 weeks	Intestine (duodenum)	Duodenal structure, oxidative stress (ROS), NF-κB/NLRP3 activation, inflammatory cytokines, tight junction proteins	PS-NPs aggravated LPS-induced duodenal inflammation and permeability via ROS-driven NF-κB/NLRP3 activation; QNZ mitigated effects	Lack of full dose/duration details; limited to duodenal outcomes	[31]
Mouse (C57BL/6)	Polypropylene microplastics (PP-MPs), 8 and 70 μm	Oral gavage, 0.1–10 mg/mL 1, 10, 100 mg/kg/day	28 days	Colon	Histopathology, redox balance, cytokines, tight junctions, apoptosis markers	PP-MPs caused oxidative stress, inflammation, apoptosis, barrier disruption via TLR4/NF-κB activation	No systemic toxicity assessment; no recovery or chronic phase	[52]
Female mouse (Balb/c)	Polystyrene nanoplastics (PS-NPs), 15 and 38 nm	Oral exposure, 1 mg/day	5 weeks	Ovary	Fertility rate, ovarian histology, apoptosis, ROS, Hippo signaling proteins	PS-NPs accumulated in ovaries, disrupted granulosa cells via ROS/Hippo signaling, reducing fertility; salidroside mitigated effects	No long-term reproductive outcomes measured	[56]
Pregnant mouse C57BL/6 (miscarriage model)	Polystyrene nanoplastics (PS-NPs), 50 nm	Oral, 50–100 mg/kg	GD 5 to GD18	Placenta and trophoblast	Oxidative stress, apoptosis, Bcl-2/caspase pathway, miscarriage incidence	PS-NPs induced miscarriage via mitochondrial apoptosis signaling; Bcl-2 overexpression mitigated effects	Human relevance inferred; unclear environmental exposure relevance	[57]
Female mouse (Kun Ming, KM)	Polystyrene nanoplastics (PS-NPs), 25 nm	Oral, 50 mg/kg (chronic exposure)	42 days	Ovary (granulosa cells, oocytes)	RNA-seq, PI3K-AKT, autophagy, apoptosis, oocyte quality	PS-NPs deactivated PI3K-AKT, triggered granulosa cell autophagy/apoptosis, reducing oocyte quality; estradiol reversed effects	No dose–response analysis; no fertility rate data	[65]
Male BALB/c mouse	Polystyrene microplastics (PS-MPs), 0.5, 4, 10 μm	Drinking water, 100 and 1000 μg/L	180 days	Testis	Hormones (T, LH, FSH), sperm quality, histology, StAR/LHR pathway	Chronic PS-MP exposure reduced testosterone and sperm quality via LHR/cAMP/PKA/StAR suppression	Only male model; environmental relevance of dose uncertain	[39]
Male Swiss albino mouse	Polystyrene microplastics (PS-MPs), 5 μm	Oral gavage, 0.1 and 0.2 mg	28 days	Thyroid	Hormones (TSH, T3, T4), oxidative stress, TSHR & TPO expression, histopathology	PS-MPs disrupted thyroid hormone balance and follicular structure via oxidative stress and gene downregulation	Only two doses; lacks systemic endocrine profiling	[66]
Female Wistar rat	Polystyrene microplastics (PS-MPs), 0.5 μm	Oral, 0.015, 0.15, 1.5 mg/day	90 days	Ovary	Follicle count, AMH, fibrosis markers, Wnt/β-catenin, oxidative stress	PS-MPs induced fibrosis via Wnt/β-catenin and ROS-driven granulosa apoptosis, reducing ovarian reserve	Lacks fertility outcome measures; limited to one particle type	[70]
Female mouse	Polystyrene microplastics (PS-MPs, 5–10 μm), 100 mg/L + DEHP 200 mg/kg	Oral (single and co-exposure), 100 mg/L	35 days	Ovary (granulosa cells)	ROS, DNA damage, Hippo & CNR1/CRBN/YY1/CYP2E1 signaling, necroptosis	Co-exposure with DEHP caused oxidative stress-mediated DNA damage, necroptosis, ovarian injury; inhibitors (AM251, DAS) reversed toxicity	Co-exposure model limits attribution of effects to MPs alone	[72]
Adult male zebrafish	Polystyrene microplastics (PS-MPs), 0.5 and 50 μm	Waterborne, 100 and 1000 μg/L	14 days	Gut microbiota	Mucus production, microbial diversity, cytokines (IL-1α, IL-1β, IFN)	PS-MPs altered gut microbiota, increased inflammation and mucus production	Aquatic model limits mammalian relevance	[81]
Male C57BL/6 mouse	Polystyrene nanoplastics (PS-NPs), unspecified nm	Oral, 30, 60, 100 mg/L	42 days	Heart	Echocardiography, blood pressure, fibrosis, TNF-α/NF-κB, P38/MAPK	PS-NPs caused ventricular dilation, fibrosis, oxidative stress, and cardiac dysfunction	Only male mice; lacks recovery or reversibility data	[114]
Male C57BL/6 Mouse	Polystyrene nanoplastics (PS-NPs), 100 nm + LPS	i.p., 5 μg/g	Every other day for 2 weeks	Heart	ROS, fibrosis markers, autophagy (AMPK/mTOR/ULK1), TGF-β/Smad	PS-NPs aggravated LPS-induced myocardial fibrosis and autophagy via ROS/TGF-β1/Smad	Lack of chronic exposure data; mechanism inferred from acute effects	[115]
Male Wistar rat	Polystyrene microplastics (PS-MPs), 0.5 μm	Oral, 0.5, 5, 50 mg/L	90 days	Heart	Serum CK-MB, troponin I, histology, Wnt/β-catenin	PS-MPs induced oxidative stress, myocardial apoptosis, fibrosis via Wnt/β-catenin	Limited mechanistic validation; male-only study	[116]
Male C57BL/6J mouse	Polystyrene nanoplastics (PS-NPs), 50 nm	Oral, 250 mg/kg/day	28 days	Brain (dopaminergic neurons)	Mitochondrial function, mitophagy (AMPK/ULK1), behavior, motor tests	PS-NPs caused PD-like neurodegeneration via excessive mitophagy; melatonin mitigated effects	High dose; short duration; only male model	[120]
Male Wistar rat	Polystyrene microplastics (PS-MPs), 500 nm	Oral, 0.015, 0.15, 1.5 mg/day	90 days	Testis	Sperm parameters, blood-testis barrier (BTB) integrity, oxidative stress, p38 MAPK/Nrf2 pathway	PS-MPs impaired spermatogenesis and BTB via MAPK-Nrf2-mediated oxidative stress	No recovery data; species differences untested	[104]

**Table 6:** Estimated Environmental Exposure vs. Experimental Dosing Across Routes of Exposure to Micro/Nanoplastics (MNPs).

Exposure Route	Estimated Human Exposure Range (Environmental, Real-World)	Typical Experimental Doses (Mechanistic Studies)	Notes on Relevance
Ingestion	~10^3^–10^5^ particles/day (≈μg–mg/day, depending on size/density) ^1^	10^6^–10^9^ particles/mL in vitro media; 10^6^–10^8^ particles/kg/day in vivo	Many in vitro doses exceed environmental estimates by several orders of magnitude; high doses often used to elicit measurable mechanistic effects.
Inhalation	~10^2^–10^4^ particles/day (higher indoors; infants may have greater exposure) ^1,2^	10^5^–10^7^ particles/mL (cell culture); acute in vivo exposures equivalent to >10^6^ particles/kg/day	Indoor air exposures generally higher than outdoor; experimental doses typically far above environmental levels.
Dermal	Quantitative estimates scarce; likely low under normal conditions ^1^	Limited studies; often high-dose topical or subcutaneous administration	Dermal uptake under environmental conditions remains poorly characterized; experimental designs may not reflect real-world exposure.

Adapted from ^1^ [133] and ^2^ [134].

## Data Availability

No new data were created or analyzed in this study. Data sharing is not applicable to this article.

## Supplementary Materials

The following supporting information can be downloaded at: https://www.mdpi.com/article/10.3390/toxics13110921/s1, File S1: Search methods.

## References

[1] Amato-Lourenço L.F., Carvalho-Oliveira R., Júnior G.R., Dos Santos Galvão L., Ando R.A., Mauad T. (2021). Presence of airborne microplastics in human lung tissue. J. Hazard. Mater..

[2] Leslie H.A., van Velzen M.J.M., Brandsma S.H., Vethaak A.D., Garcia-Vallejo J.J., Lamoree M.H. (2022). Discovery and quantification of plastic particle pollution in human blood. Environ. Int..

[3] Ragusa A., Svelato A., Santacroce C., Catalano P., Notarstefano V., Carnevali O., Papa F., Rongioletti M.C.A., Baiocco F., Draghi S. (2021). Plasticenta: First evidence of microplastics in human placenta. Environ. Int..

[4] Schwabl P., Köppel S., Königshofer P., Bucsics T., Trauner M., Reiberger T., Liebmann B. (2019). Detection of Various Microplastics in Human Stool: A Prospective Case Series. Ann. Intern. Med..

[5] Bhagat J., Nishimura N., Shimada Y. (2021). Toxicological interactions of microplastics/nanoplastics and environmental contaminants: Current knowledge and future perspectives. J. Hazard. Mater..

[6] Hu L., Zhao Y., Xu H. (2022). Trojan horse in the intestine: A review on the biotoxicity of microplastics combined environmental contaminants. J. Hazard. Mater..

[7] Rochman C.M., Hoh E., Hentschel B.T., Kaye S. (2013). Long-term field measurement of sorption of organic contaminants to five types of plastic pellets: Implications for plastic marine debris. Environ. Sci. Technol..

[8] Walczak A.P., Kramer E., Hendriksen P.J., Tromp P., Helsper J.P., van der Zande M., Rietjens I.M., Bouwmeester H. (2015). Translocation of differently sized and charged polystyrene nanoparticles in in vitro intestinal cell models of increasing complexity. Nanotoxicology.

[9] WHO (World Health Organization) (2019). Microplastics in Drinking-Water.

[10] EFSA Panel on Contaminants in the Food Chain (CONTAM) (2016). Presence of microplastics and nanoplastics in food, with particular focus on seafood. EFSA J. Eur. Food Saf. Auth..

[11] Araújo A.M., Mota C., Ramos H., Faria M.A., Carvalho M., Ferreira I.M.P.L.V.O. (2025). The neurotoxic threat of micro- and nanoplastics: Evidence from In Vitro and In Vivo models. Arch. Toxicol..

[12] Yee M.S., Hii L.W., Looi C.K., Lim W.M., Wong S.F., Kok Y.Y., Tan B.K., Wong C.Y., Leong C.O. (2021). Impact of Microplastics and Nanoplastics on Human Health. Nanomaterials.

[13] Murphy F.A., Poland C.A., Duffin R., Al-Jamal K.T., Ali-Boucetta H., Nunes A., Byrne F., Prina-Mello A., Volkov Y., Li S. (2011). Length-dependent retention of carbon nanotubes in the pleural space of mice initiates sustained inflammation and progressive fibrosis on the parietal pleura. Am. J. Pathol..

[14] Champion J.A., Mitragotri S. (2006). Role of target geometry in phagocytosis. Proc. Natl. Acad. Sci. USA.

[15] Arezki Y., Delalande F., Schaeffer-Reiss C., Cianférani S., Rapp M., Lebeau L., Pons F., Ronzani C. (2022). Surface charge influences protein corona, cell uptake and biological effects of carbon dots. Nanoscale.

[16] Cao J., Yang Q., Jiang J., Dalu T., Kadushkin A., Singh J., Fakhrullin R., Wang F., Cai X., Li R. (2022). Coronas of micro/nano plastics: A key determinant in their risk assessments. Part. Fibre Toxicol..

[17] Joksimovic N., Selakovic D., Jovicic N., Jankovic N., Pradeepkumar P., Eftekhari A., Rosic G. (2022). Nanoplastics as an invisible threat to humans and the environment. J. Nanomater..

[18] Foroozandeh P., Aziz A.A. (2018). Insight into Cellular Uptake and Intracellular Trafficking of Nanoparticles. Nanoscale Res. Lett..

[19] Ullah S., Ahmad S., Guo X., Ullah S., Ullah S., Nabi G., Wanghe K. (2023). A review of the endocrine disrupting effects of micro and nano plastic and their associated chemicals in mammals. Front. Endocrinol..

[20] Casella C., Ballaz S.J. (2024). Genotoxic and neurotoxic potential of intracellular nanoplastics: A review. J. Appl. Toxicol..

[21] Ruan Y., Zhong Z., Liu X., Li Z., Li J., Sun L., Sen H. (2023). Correlation between cellular uptake and cytotoxicity of polystyrene micro/nanoplastics in HeLa cells: A size-dependent matter. PLoS ONE.

[22] Bai C.L., Wang D., Luan Y.L., Huang S.N., Liu L.Y., Guo Y. (2024). A review on micro- and nanoplastics in humans: Implication for their translocation of barriers and potential health effects. Chemosphere.

[23] Rajendran D., Chandrasekaran N. (2023). Journey of micronanoplastics with blood components. RSC Adv..

[24] Martin L., Simpson K., Brzezinski M., Watt J., Xu W. (2024). Cellular response of keratinocytes to the entry and accumulation of nanoplastic particles. Part. Fibre Toxicol..

[25] Zhang T., Wang Z., Wu Y., Zhu S., Su J. (2025). Interactions of micro- and nanoplastics with biomolecules: From public health to protein corona effect and beyond. J. Phys. Chem. B.

[26] Basini G., Grolli S., Bertini S., Bussolati S., Berni M., Berni P., Ramoni R., Scaltriti E., Quintavalla F., Grasselli F. (2023). Nanoplastics induced oxidative stress and VEGF production in aortic endothelial cells. Environ. Toxicol. Pharmacol..

[27] Zhang H., Cheng H., Wang Y., Duan Z., Cui W., Shi Y., Qin L. (2022). Influence of functional group modification on the toxicity of nanoplastics. Front. Mar. Sci..

[28] Das A. (2023). The emerging role of microplastics in systemic toxicity: Involvement of reactive oxygen species (ROS). Sci. Total Environ..

[29] Kadac-Czapska K., Ośko J., Knez E., Grembecka M. (2024). Microplastics and Oxidative Stress-Current Problems and Prospects. Antioxidants.

[30] Li Y., Guo M., Niu S., Shang M., Chang X., Sun Z., Zhang R., Shen X., Xue Y. (2023). ROS and DRP1 interactions accelerate the mitochondrial injury induced by polystyrene nanoplastics in human liver HepG2 cells. Chem.-Biol. Interact..

[31] He Y., Li Z., Xu T., Luo D., Chi Q., Zhang Y., Li S. (2022). Polystyrene nanoplastics deteriorate LPS-modulated duodenal permeability and inflammation in mice via ROS drived-NF-κB/NLRP3 pathway. Chemosphere.

[32] Mahmud F., Sarker D.B., Jocelyn J.A., Sang Q.A. (2024). Molecular and Cellular Effects of Microplastics and Nanoplastics: Focus on Inflammation and Senescence. Cells.

[33] Panizzolo M., Martins V.H., Ghelli F., Squillacioti G., Bellisario V., Garzaro G., Bosio D., Colombi N., Bono R., Bergamaschi E. (2023). Biomarkers of oxidative stress, inflammation, and genotoxicity to assess exposure to micro- and nanoplastics. A literature review. Ecotoxicol. Environ. Saf..

[34] Jayavel S., Govindaraju B., Michael J.R., Viswanathan B. (2024). Impacts of micro and nanoplastics on human health. Bull. Natl. Res. Cent..

[35] Lin S., Zhang H., Wang C., Su X.L., Song Y., Wu P., Yang Z., Wong M.H., Cai Z., Zheng C. (2022). Metabolomics Reveal Nanoplastic-Induced Mitochondrial Damage in Human Liver and Lung Cells. Environ. Sci. Technol..

[36] Wang K., Du Y., Li P., Guan C., Zhou M., Wu L., Liu Z., Huang Z. (2024). Nanoplastics causes heart aging/myocardial cell senescence through the Ca^2+^/mtDNA/cGAS-STING signaling cascade. J. Nanobiotechnol..

[37] Kustra A., Zając M., Bednarczyk P., Maliszewska-Olejniczak K. (2025). Exposure to polystyrene nanoparticles leads to dysfunction in DNA repair mechanisms in Caco-2 cells. Biol. Res..

[38] Ojo A.B., Agbeye O.D., Ogwa T.O., Adedoyin D., Rotimi D.E., Ojo O.A. (2025). Implications of plastic-derived endocrine disruptors on human health. Toxicol. Mech. Methods.

[39] Jin H., Yan M., Pan C., Liu Z., Sha X., Jiang C., Li L., Pan M., Li D., Han X. (2022). Chronic exposure to polystyrene microplastics induced male reproductive toxicity and decreased testosterone levels via the LH-mediated LHR/cAMP/PKA/StAR pathway. Part. Fibre Toxicol..

[40] Saha U., Kumari P., Ghosh A., Sinha A., Jena S., Kirti A., Gupta A., Choudhury A., Simnani F.Z., Nandi A. (2024). Detrimental consequences of micropolymers associated plasticizers on endocrinal disruption. Mater. Today Bio.

[41] Najahi H., Alessio N., Venditti M., Oliveri Conti G., Ferrante M., Di Bernardo G., Galderisi U., Minucci S., Banni M. (2025). Impact of Environmental Microplastic Exposure on Caco-2 Cells: Unraveling Proliferation, Apoptosis, and Autophagy Activation. Int. J. Environ. Res. Public Health.

[42] Fanghella F., Pesce M., Franceschelli S., Panella V., Elsallabi O., Lupi T., Rizza B., Di Battista M.G., Bruno A., Ballerini P. (2025). Biological modulation of autophagy by nanoplastics: A current overview. Int. J. Mol. Sci..

[43] Ma Y., Yu J., Sun J., Zhu Y., Li X., Liu X., Zhang X., Liu L., Li L., Yang J. (2025). Dust Fall Microplastics from a Megacity of China Inhibit Autophagy via the PI3K/Akt/mTOR Pathway. Environ. Health.

[44] He Y., Yu T., Li H., Sun Q., Chen M., Lin Y., Dai J., Wang W., Li Q., Ju S. (2024). Polystyrene nanoplastic exposure actives ferroptosis by oxidative stress-induced lipid peroxidation in porcine oocytes during maturation. J. Anim. Sci. Biotechnol..

[45] Fu X., Han H., Yang H., Xu B., Dai W., Liu L., He T., Du X., Pei X. (2024). Nrf2-mediated ferroptosis of spermatogenic cells involved in male reproductive toxicity induced by polystyrene nanoplastics in mice. J. Zhejiang Univ.–Sci. B.

[46] Zhao H., Wang Z., Wang H. (2025). The role of NCOA4-mediated ferritinophagy in the ferroptosis of hepatocytes: A mechanistic viewpoint. Pathol. Res. Pract..

[47] Qiao J., Chen R., Wang M., Bai R., Cui X., Liu Y., Wu C., Chen C. (2021). Perturbation of gut microbiota plays an important role in micro/nanoplastics-induced gut barrier dysfunction. Nanoscale.

[48] Bora S.S., Gogoi R., Sharma M.R., Anshu Borah M.P., Deka P., Bora J., Naorem R.S., Das J., Teli A.B. (2024). Microplastics and human health: Unveiling the gut microbiome disruption and chronic disease risks. Front. Cell. Infect. Microbiol..

[49] Ullah H., Arbab S., Tian Y., Liu C.Q., Chen Y., Qijie L., Khan M.I.U., Hassan I.U., Li K. (2023). The gut microbiota-brain axis in neurological disorder. Front. Neurosci..

[50] Dal Yöntem F., Aydoğan Ahbab M. (2024). Mitochondria as a Target of Micro- and Nanoplastic Toxicity.

[51] Cortés C., Domenech J., Salazar M., Pastor S., Marcos R., Hernández A. (2020). Nanoplastics as a potential environmental health factor: Effects of polystyrene nanoparticles on human intestinal epithelial Caco-2 cells. Environ. Sci. Nano.

[52] Jia R., Han J., Liu X., Li K., Lai W., Bian L., Yan J., Xi Z. (2023). Exposure to Polypropylene Microplastics via Oral Ingestion Induces Colonic Apoptosis and Intestinal Barrier Damage through Oxidative Stress and Inflammation in Mice. Toxics.

[53] Weber A., Schwiebs A., Solhaug H., Stenvik J., Nilsen A.M., Wagner M., Relja B., Radeke H.H. (2022). Nanoplastics affect the inflammatory cytokine release by primary human monocytes and dendritic cells. Environ. Int..

[54] Bishop B., Webber W.S., Atif S.M., Ley A., Pankratz K.A., Kostelecky R., Colgan S.P., Dinarello C.A., Zhang W., Li S. (2025). Micro- and nano-plastics induce inflammation and cell death in human cells. Front. Immunol..

[55] Wu D., Zhang M., Bao T.T., Lan H. (2023). Long-term exposure to polystyrene microplastics triggers premature testicular aging. Part. Fibre Toxicol..

[56] Zeng L., Zhou C., Xu W., Huang Y., Wang W., Ma Z., Huang J., Li J., Hu L., Xue Y. (2023). The ovarian-related effects of polystyrene nanoplastics on human ovarian granulosa cells and female mice. Ecotoxicol. Environ. Saf..

[57] Wan S., Wang X., Chen W., Wang M., Zhao J., Xu Z., Wang R., Mi C., Zheng Z., Zhang H. (2024). Exposure to high dose of polystyrene nanoplastics causes trophoblast cell apoptosis and induces miscarriage. Part. Fibre Toxicol..

[58] Ahmadi P., Doyle D., Mojarad N., Taherkhani S., Janzadeh A., Honardoost M., Gholami M. (2025). Effects of micro- and nanoplastic exposure on macrophages: A review of molecular and cellular mechanisms. Toxicol. Mech. Methods.

[59] Hirt N., Body-Malapel M. (2020). Immunotoxicity and intestinal effects of nano- and microplastics: A review of the literature. Part. Fibre Toxicol..

[60] Zhu X., Wang C., Duan X., Liang B., Genbo Xu E., Huang Z. (2023). Micro- and nanoplastics: A new cardiovascular risk factor?. Environ. Int..

[61] Nihart A.J., Garcia M.A., El Hayek E., Liu R., Olewine M., Kingston J.D., Castillo E.F., Gullapalli R.R., Howard T., Bleske B. (2025). Bioaccumulation of microplastics in decedent human brains. Nat. Med..

[62] Berber A.A., Akinci Kenanoğlu N., Nur Demi R.Ş., Aksoy H. (2025). Genotoxic and cytotoxic effects of polystyrene nanoplastics on human lymphocytes: A comprehensive analysis. Mutat. Res. Genet. Toxicol. Environ. Mutagen..

[63] Płuciennik K., Sicińska P., Misztal W., Bukowska B. (2024). Important Factors Affecting Induction of Cell Death, Oxidative Stress and DNA Damage by Nano- and Microplastic Particles In Vitro. Cells.

[64] Møller P., Roursgaard M. (2023). Exposure to nanoplastic particles and DNA damage in mammalian cells. Mutat. Res. Rev. Mutat. Res..

[65] Xue Y., Cheng X., Ma Z.Q., Wang H.P., Zhou C., Li J., Zhang D.L., Hu L.L., Cui Y.F., Huang J. (2024). Polystyrene nanoplastics induce apoptosis, autophagy, and steroidogenesis disruption in granulosa cells to reduce oocyte quality and fertility by inhibiting the PI3K/AKT pathway in female mice. J. Nanobiotechnol..

[66] Islam M.S., Kamruzzaman M., Rima U.K. (2025). Polystyrene Microplastics-Induced Thyroid Dysfunction in Mice: A Study of Gene Expression, Oxidative Stress, and Histopathological Changes. Vet. Med. Sci..

[67] Wang Z., Wang Y., Zhang J., Feng G., Miao S., Lu R., Tian X., Ye Y. (2025). Antioxidant intervention against microplastic hazards. Antioxidants.

[68] Aloisi M., Poma A.M.G. (2025). Nanoplastics as Gene and Epigenetic Modulators of Endocrine Functions: A Perspective. Int. J. Mol. Sci..

[69] Tyc H.J., Kłodnicka K., Teresińska B., Karpiński R., Flieger J., Baj J. (2025). Micro- and Nanoplastics as Disruptors of the Endocrine System-A Review of the Threats and Consequences Associated with Plastic Exposure. Int. J. Mol. Sci..

[70] An R., Wang X., Yang L., Zhang J., Wang N., Xu F., Hou Y., Zhang H., Zhang L. (2021). Polystyrene microplastics cause granulosa cells apoptosis and fibrosis in ovary through oxidative stress in rats. Toxicology.

[71] Zhang A., Wang Y., Xue Q., Yao J., Chen L., Feng S., Shao J., Guo Z., Zhou B., Xie J. (2025). Polystyrene microplastics disrupt kidney organoid development via oxidative stress and Bcl-2/Bax/caspase pathway. Chem. Biol. Interact..

[72] Wu H., Liu Q., Yang N., Xu S. (2023). Polystyrene-microplastics and DEHP co-exposure induced DNA damage, cell cycle arrest and necroptosis of ovarian granulosa cells in mice by promoting ROS production. Sci. Total Environ..

[73] Mu Y., Sun J., Li Z., Zhang W., Liu Z., Li C., Peng C., Cui G., Shao H., Du Z. (2022). Activation of pyroptosis and ferroptosis is involved in the hepatotoxicity induced by polystyrene microplastics in mice. Chemosphere.

[74] Bu W., Cui Y., Jin Y., Wang X., Jiang M., Huang R., Egbobe J.O., Zhao X., Tang J. (2024). Unmasking the Invisible Threat: Biological Impacts and Mechanisms of Polystyrene Nanoplastics on Cells. Toxics.

[75] Wu Y., Wang J., Zhao T., Sun M., Xu M., Che S., Pan Z., Wu C., Shen L. (2024). Polystyrenenanoplastics lead to ferroptosis in the lungs. J. Adv. Res..

[76] Wang L., Zhang X., Xu M., Zheng G., Chen J., Li S., Cui J., Zhang S. (2023). Implication of ferroptosis in hepatic toxicity upon single or combined exposure to polystyrene microplastics and cadmium. Environ. Pollut. (Barking Essex 1987).

[77] Yu H., Zhao Z., Li H., Han Y., Li H., Cui C., Hu Y., Zhang B. (2025). Nanoplastics exposure exacerbates Aβ plaque deposition in Alzheimer’s disease mice by inducing microglia pyroptosis. Ecotoxicol. Environ. Saf..

[78] Li G., Liu X., Sun X., Huang L., Kuang W., Ou J., Zhang J., Zhang Z., Li H., Tang H. (2024). Polystyrene microplastics induce anxiety via HRAS derived PERK-NF-κB pathway. Environ. Int..

[79] Tang Q., Li T., Chen K., Deng X., Zhang Q., Tang H., Shi Z., Zhu T., Zhu J. (2022). PS-NPs Induced Neurotoxic Effects in SHSY-5Y Cells via Autophagy Activation and Mitochondrial Dysfunction. Brain Sci..

[80] Demarquoy J. (2024). Microplastics and microbiota: Unraveling the hidden environmental challenge. World J. Gastroenterol..

[81] Jin Y., Xia J., Pan Z., Yang J., Wang W., Fu Z. (2018). Polystyrene microplastics induce microbiota dysbiosis and inflammation in the gut of adult zebrafish. Environ. Pollut. (Barking Essex 1987).

[82] Ghosh A., Gorain B. (2025). Mechanistic insight of neurodegeneration due to micro/nano-plastic-induced gut dysbiosis. Arch. Toxicol..

[83] Peng Y., Lu J., Fan L., Dong W., Jiang M. (2024). Simulated gastrointestinal digestion of two different sources of biodegradable microplastics and the influence on gut microbiota. Food Chem. Toxicol..

[84] Zheng P.C., Li R., Lai K.P., Zhang X.X. (2024). Biological exposure to microplastics and nanoplastics and plastic additives: Impairment of glycolipid metabolism and adverse effects on metabolic diseases. Environ. Sci. Pollut. Res. Int..

[85] Chi J., Patterson J.S., Jin Y., Kim K.J., Lalime N., Hawley D., Lewis F., Li L., Wang X., Campen M.J. (2025). Metabolic Reprogramming in Gut Microbiota Exposed to Polystyrene Microplastics. Biomedicines.

[86] Fackelmann G., Sommer S. (2019). Microplastics and the gut microbiome: How chronically exposed species may suffer from gut dysbiosis. Mar. Pollut. Bull..

[87] Elisso M.C., Billè B., Cappello T., Maisano M. (2024). Polystyrene micro- and nanoplastics (PS MNPs): A review of recent advances in the use of -omics in PS MNP toxicity studies on aquatic organisms. Fishes.

[88] Limonta G., Mancia A., Benkhalqui A., Bertolucci C., Abelli L., Fossi M.C., Panti C. (2019). Microplastics induce transcriptional changes, immune response and behavioral alterations in adult zebrafish. Sci. Rep..

[89] Wang X., Zhao J., Gao M., Wang T., Zhang H. (2025). Mechanism of nano-plastics induced inflammation injury in vascular endothelial cells. J. Environ. Sci..

[90] Capolupo M., Sørensen L., Jayasena K.D.R., Booth A.M., Fabbri E. (2020). Chemical composition and ecotoxicity of plastic and car tire rubber leachates to aquatic organisms. Water Res..

[91] Marycleopha M., Balarabe B.Y., Kumar S., Adjama I. (2025). Exploring the impact of microplastics and nanoplastics on macromolecular structure and functions. J. Appl. Toxicol..

[92] Covello C., Di Vincenzo F., Cammarota G., Pizzoferrato M. (2024). Micro(nano)plastics and Their Potential Impact on Human Gut Health: A Narrative Review. Curr. Issues Mol. Biol..

[93] Ma J., Ladd D.M., Kaval N., Wang H.S. (2025). Toxicity of long term exposure to low dose polystyrene microplastics and nanoplastics in human iPSC-derived cardiomyocytes. Food Chem. Toxicol..

[94] Huang F., You H., Tang X., Su Y., Peng H., Li H., Wei Z., Hua J. (2025). Early-life exposure to polypropylene nanoplastics induces neurodevelopmental toxicity in mice and human iPSC-derived cerebral organoids. J. Nanobiotechnol..

[95] de Sousa A.K.A., Pires K.S.N., Cavalcante I.H., Cavalcante I.C.L., Santos J.D., Queiroz M.I.C., Leite A.C.R., Crispim A.C., da Rocha Junior E.R., Aquino T.M. (2024). Polystyrene microplastics exposition on human placental explants induces time-dependent cytotoxicity, oxidative stress and metabolic alterations. Front. Endocrinol..

[96] Hwang J., Choi D., Han S., Choi J., Hong J. (2019). An assessment of the toxicity of polypropylene microplastics in human derived cells. Sci. Total Environ..

[97] Djouina M., Loison S., Body-Malapel M. (2024). Recent progress in intestinal toxicity of microplastics and nanoplastics: Systematic review of preclinical evidence. Microplastics.

[98] Lee S.E., Kim D.Y., Jeong T.S., Park Y.S. (2025). Micro- and Nano-Plastic-Induced Adverse Health Effects on Lungs and Kidneys Linked to Oxidative Stress and Inflammation. Life.

[99] Xu X., Goros R.A., Dong Z., Meng X., Li G., Chen W., Liu S., Ma J., Zuo Y.Y. (2023). Microplastics and Nanoplastics Impair the Biophysical Function of Pulmonary Surfactant by Forming Heteroaggregates at the Alveolar-Capillary Interface. Environ. Sci. Technol..

[100] Xue D., Huang J., Sun X., Zhang W., Ma H., Yin D., Wang Y., Wang J., Yang C., Geng Q. (2025). Dissection of the potential mechanism of polystyrene microplastic exposure on cardiomyocytes. Sci. Total Environ..

[101] Zhong Y., Feng Y., Huang Y., Wang B., Shi W., Liang B., Li Z., Zhang B., Du J., Xiu J. (2024). Polystyrene nanoplastics accelerate atherosclerosis: Unraveling the impact on smooth muscle cells through KIF15-mediated migration. Ecotoxicol. Environ. Saf..

[102] Kumari S., Begum M.Y., Chinglenthoiba C., Aran K.R., Panda S.P., Abomughaid M.M., Lakhanpal S., Avinash D., Jha N.K., Gupta R. (2025). Deciphering the Neurotoxic Burden of Micro- and Nanoplastics: From Multi-model Experimental Evidence to Therapeutic Innovation. Mol. Neurobiol..

[103] Camerano Spelta Rapini C., Di Berardino C., Peserico A., Capacchietti G., Barboni B. (2024). Can Mammalian Reproductive Health Withstand Massive Exposure to Polystyrene Micro- and Nanoplastic Derivatives? A Systematic Review. Int. J. Mol. Sci..

[104] Li S., Wang Q., Yu H., Yang L., Sun Y., Xu N., Wang N., Lei Z., Hou J., Jin Y. (2021). Polystyrene microplastics induce blood-testis barrier disruption regulated by the MAPK-Nrf2 signaling pathway in rats. Environ. Sci. Pollut. Res. Int..

[105] Chen K., Wang L., Liu J., Zheng H., Wu X., Liao X. (2025). The ant that may well destroy a whole dam: A systematic review of the health implication of nanoplastics/microplastics through gut microbiota. Crit. Rev. Food Sci. Nutr..

[106] Severino A., Tohumcu E., Tamai L., Dargenio P., Porcari S., Rondinella D., Venturini I., Maida M., Gasbarrini A., Cammarota G. (2024). The microbiome-driven impact of western diet in the development of noncommunicable chronic disorders. Best Pract. Res. Clin. Gastroenterol..

[107] Shen Y., Fan N., Ma S.X., Cheng X., Yang X., Wang G. (2025). Gut Microbiota Dysbiosis: Pathogenesis, Diseases, Prevention, and Therapy. MedComm.

[108] Zhao H., Lin G., Yin Y., Wu Q., Wang Y., Tang N., Qi X. (2025). Impact of micro- and nanoplastics on gastrointestinal diseases: Recent advances. Eur. J. Intern. Med..

[109] Cheng Y., Chen J., Fu R., Zhang P., Chen H., Cao H., Jiang Z., Hong Y., Li Y., He C. (2025). Molecular mechanism differences between nanoplastics and microplastics in colon toxicity: Nanoplastics induce ferroptosis-mediated immunogenic cell death, while microplastics cause cell metabolic reprogramming. J. Nanobiotechnol..

[110] Zeyneloglu C., Babayev H., Ogulur I., Ardicli S., Pat Y., Yazici D., Zhao B., Chang L., Liu X., D’Avino P. (2025). The epithelial barrier theory proposes a comprehensive explanation for the origins of allergic and other chronic noncommunicable diseases. FEBS Lett..

[111] Chung K.F., Adcock I.M. (2008). Multifaceted mechanisms in COPD: Inflammation, immunity, and tissue repair and destruction. Eur. Respir. J..

[112] Liu G., Bao Q., Zhang C., Zhong Y., Deng M., Huang Y., Ye Z., Jing J. (2025). PVC nanoplastics impair cardiac function via lysosomal and mitochondrial dysfunction. Biochem. Biophys. Res. Commun..

[113] Zhang T., Yang S., Ge Y., Yin L., Pu Y., Gu Z., Chen Z., Liang G. (2024). Unveiling the Heart’s Hidden Enemy: Dynamic Insights into Polystyrene Nanoplastic-Induced Cardiotoxicity Based on Cardiac Organoid-on-a-Chip. ACS Nano.

[114] Xiong Z., Kong Q., Hua J., Chen Q., Wang D. (2025). Cardiotoxicity of polystyrene nanoplastics and associated mechanism of myocardial cell injury in mice. Ecotoxicol. Environ. Saf..

[115] Lin P., Tong X., Xue F., Qianru C., Xinyu T., Zhe L., Zhikun B., Shu L. (2022). Polystyrene nanoplastics exacerbate lipopolysaccharide-induced myocardial fibrosis and autophagy in mice via ROS/TGF-β1/Smad. Toxicology.

[116] Li Z., Zhu S., Liu Q., Wei J., Jin Y., Wang X., Zhang L. (2020). Polystyrene microplastics cause cardiac fibrosis by activating Wnt/β-catenin signaling pathway and promoting cardiomyocyte apoptosis in rats. Environ. Pollut..

[117] Chen J., Yan L., Zhang Y., Liu X., Wei Y., Zhao Y., Li K., Shi Y., Liu H., Lai W. (2024). Maternal exposure to nanopolystyrene induces neurotoxicity in offspring through P53-mediated ferritinophagy and ferroptosis in the rat hippocampus. J. Nanobiotechnol..

[118] Chen Y., Nan Y., Xu L., Dai A., Orteg R.M.M., Ma M., Zeng Y., Li J. (2025). Polystyrene nanoplastics exposure induces cognitive impairment in mice via induction of oxidative stress and ERK/MAPK-mediated neuronal cuproptosis. Part. Fibre Toxicol..

[119] Suman A., Mahapatra A., Gupta P., Ray S.S., Singh R.K. (2023). Polystyrene microplastics modulated bdnf expression triggering neurotoxicity via apoptotic pathway in zebrafish embryos. Comparative biochemistry and physiology. Comp. Biochem. Physiol. C Toxicol. Pharmacol..

[120] Huang Y., Liang B., Li Z., Zhong Y., Wang B., Zhang B., Du J., Ye R., Xian H., Min W. (2023). Polystyrene nanoplastic exposure induces excessive mitophagy by activating AMPK/ULK1 pathway in differentiated SH-SY5Y cells and dopaminergic neurons in vivo. Part. Fibre Toxicol..

[121] Lafram A., Krami M., Akarid K., Laadraoui J., Roky R. (2024). Effects of exposure to micro/nanoplastics of polystyrene on neuronal oxidative stress, neuroinflammation, and anxiety-like behavior in mice: A systematic review. Emerg. Contam..

[122] Budhwar M., Mehra S., Sharma M., Ahsan A.U., Chopra M. (2025). Unveiling micro-nanoplastics (MNPs) induced developmental toxicity, transgenerational transport and associated signaling pathways. J. Hazard. Mater. Adv..

[123] He Y., Yin R. (2024). The reproductive and transgenerational toxicity of microplastics and nanoplastics: A threat to mammalian fertility in both sexes. J. Appl. Toxicol..

[124] Yu F., Qin Q., Zhang X., Ma J. (2024). Characteristics and adsorption behavior of typical microplastics in long-term accelerated weathering simulation. Environ. Sci. Process. Impacts.

[125] Hartmann N.B., Hüffer T., Thompson R.C., Hassellöv M., Verschoor A., Daugaard A.E., Rist S., Karlsson T., Brennholt N., Cole M. (2019). Are We Speaking the Same Language? Recommendations for a Definition and Categorization Framework for Plastic Debris. Environ. Sci. Technol..

[126] Cedervall T., Lynch I., Lindman S., Berggård T., Thulin E., Nilsson H., Dawson K.A., Linse S. (2007). Understanding the nanoparticle-protein corona using methods to quantify exchange rates and affinities of proteins for nanoparticles. Proc. Natl. Acad. Sci. USA.

[127] Koelmans A.A., Besseling E., Shim W.J., Bergmann M., Gutow L., Klages M. (2015). Nanoplastics in the Aquatic Environment. Marine Anthropogenic Litter.

[128] Gigault J., El Hadri H., Nguyen B., Grassl B., Rowenczyk L., Tufenkji N., Feng S., Wiesner M. (2021). Nanoplastics are neither microplastics nor engineered nanoparticles. Nat. Nanotechnol..

[129] Hassoun A., Pasti L., Chenet T., Rusanova P., Smaoui S., Aït-Kaddour A., Bono G. (2023). Detection methods of micro and nanoplastics. Adv. Food Nutr. Res..

[130] Adhikari S., Kelkar V., Kumar R., Halden R.U. (2022). Methods and challenges in the detection of microplastics and nanoplastics: A mini-review. Polym. Int..

[131] Domenech J., Marcos R. (2021). Pathways of human exposure to microplastics, and estimation of the total burden. Curr. Opin. Food Sci..

[132] WHO (World Health Organization) (2022). Dietary and Inhalation Exposure to Nano- and Microplastic Particles and Potential Implications for Human Health.

[133] Zarus G.M., Muianga C., Hunter C.M., Pappas R.S. (2021). A review of data for quantifying human exposures to micro and nanoplastics and potential health risks. Sci. Total Environ..

[134] Eberhard T., Casillas G., Zarus G.M., Barr D.B. (2024). Systematic review of microplastics and nanoplastics in indoor and outdoor air: Identifying a framework and data needs for quantifying human inhalation exposures. J. Expo. Sci. Environ. Epidemiol..

[135] Zimmermann L., Dierkes G., Ternes T.A., Völker C., Wagner M. (2019). Benchmarking the in Vitro Toxicity and Chemical Composition of Plastic Consumer Products. Environ. Sci. Technol..

[136] Käppler A., Windrich F., Löder M.G., Malanin M., Fischer D., Labrenz M., Eichhorn K.J., Voit B. (2015). Identification of microplastics by FTIR and Raman microscopy: A novel silicon filter substrate opens the important spectral range below 1300 cm^−1^ for FTIR transmission measurements. Anal. Bioanal. Chem..

[137] Primpke S., Fischer M., Lorenz C., Gerdts G., Scholz-Böttcher B.M. (2020). Comparison of pyrolysis gas chromatography/mass spectrometry and hyperspectral FTIR imaging spectroscopy for the analysis of microplastics. Anal. Bioanal. Chem..

[138] Rauert C., Charlton N., Bagley A., Dunlop S.A., Symeonides C., Thomas K.V. (2025). Assessing the Efficacy of Pyrolysis-Gas Chromatography-Mass Spectrometry for Nanoplastic and Microplastic Analysis in Human Blood. Environ. Sci. Technol..

[139] Valido I.H., Fuentes-Cebrian V., Hernández A., Valiente M., López-Mesas M. (2023). Validated method for polystyrene nanoplastic separation in aqueous matrices by asymmetric-flow field flow fraction coupled to MALS and UV-Vis detectors. Mikrochimica Acta.

[140] Loeschner K., Vidmar J., Hartmann N.B., Bienfait A.M., Velimirovic M. (2023). Finding the tiny plastic needle in the haystack: How field flow fractionation can help to analyze nanoplastics in food. Anal. Bioanal. Chem..

[141] Nene A., Sadeghzade S., Viaroli S., Yang W., Uchenna U.P., Kandwal A., Liu X., Somani P., Galluzzi M. (2025). Recent advances and future technologies in nano-microplastics detection. Environ. Sci. Eur..

[142] Wang L., Ma J.Q., Song L.J., Qu X.P., Zhang Y., Fan H.M., Wang C., Zheng L.L., Gao G.D., Qu Y. (2025). Comprehensive multi-omics, behavioral and morphological analysis of the hazards of nano-plastics in mice with internal carotid artery occlusion. Ecotoxicol. Environ. Saf..

[143] Garrido Gamarro E., Costanzo V. (2022). Microplastics in Food Commodities—A Food Safety Review on Human Exposure Through Dietary Sources (Food Safety and Quality Series No. 18).

[144] Barrowclough D., Deere Birkbeck C. (2022). Transforming the Global Plastics Economy: The Role of Economic Policies in the Global Governance of Plastic Pollution. Soc. Sci..

[145] Nielsen M.B., Clausen L.P.W., Cronin R., Hansen S.F., Oturai N.G., Syberg K. (2023). Unfolding the science behind policy initiatives targeting plastic pollution. Microplastics Nanoplastics.

[146] OECD (2024). Policy Scenarios for Eliminating Plastic Pollution by 2040. Organisation for Economic Co-operation and Development..

