# Supplementary material for: Cellular and Molecular Mechanisms of Micro- and Nanoplastics Driving Adverse Human Health Effects
Source: Toxics. 2025 Oct 28;13(11):921. doi: 10.3390/toxics13110921 (PMC12656411; doi:10.3390/toxics13110921)
Supplement: Supplementary file 1 [file toxics-13-00921-s001.zip › toxics-3864115-supplementary.pdf]

Review

Cellular and Molecular Mechanisms of Micro- and Nanoplastics Driving Adverse Human Health Effects

Antonio F. Hernández [1,2,3,*], Marina Lacasaña [2,3,4,5], Aristidis M. Tsatsakis [6,7,8] and Anca Oana Docea [9]

[1] Department of Legal Medicine and Toxicology, School of Medicine, University of Granada, 18016 Granada, Spain
[2] Health Research Institute of Granada (Instituto Biosanitario de Granada, Ibs.GRANADA), 18012 Granada, Spain; marina.lacasana.easp@juntadeandalucia.es
[3] Consortium for Biomedical Research in Epidemiology and Public Health (CIBERESP), 28029 Madrid, Spain
[4] Andalusian School of Public Health (EASP), 18011 Granada, Spain
[5] Andalusian Health and Environment Observatory (OSMAN), 18011 Granada, Spain
[6] Center of Toxicology & Science Applications, Medical School, University of Crete, 71003 Heraklion, Greece; tsatsaka@uoc.gr
[7] Biomedical Science and Technology Park, Universidad ECOTEC, Km 13.5 Samborondón, Samborondon 092302, Ecuador
[8] Human Development and Health Science Faculty, Sechenov I.M., First State Medical University, 119991 Moscow, Russia
[9] Department of Toxicology, University of Medicine and Pharmacy of Craiova, 200349 Craiova, Romania; daoana00@gmail.com
* Correspondence: ajerez@ugr.es; Tel.: +34-958-249-927

Supplementary Material

Search methods

This narrative review was informed by targeted literature searches in PubMed/MEDLINE, supplemented by citation-tracking. Study inclusion was based on expert judgment to ensure coverage of the most relevant mechanistic evidence. Search terms included combinations of *"microplastic," "nanoplastic," "micro- and nanoplastics," "polystyrene," "toxicity," "oxidative stress," "inflammation," "placenta," "blood-brain barrier,"* and related keywords. Searches covered publications through July 2025 (final cut-off date to be specified in the revised submission).

We included original human observational and biomonitoring studies, in vivo mechanistic animal studies, and in vitro mechanistic studies reporting both physicochemical characterization of particles and biological endpoints. Non-English papers were considered when an English abstract or full text was available. Because the aim was to provide a mechanistic synthesis across diverse experimental platforms rather than to conduct an exhaustive meta-analysis of prevalence or exposure, we deliberately adopted a narrative approach.

To enhance transparency, we also conducted focused PubMed searches in July 2025 using structured strings designed to capture systemic (non-organ-specific) and organ-specific effects. Examples include:

1. **Systemic effects:**
   - (microplastic OR nanoplastics) AND (oxidative stress) AND mechanis* AND toxic* AND human – (253 hits)
   - (microplastic OR nanoplastics) AND inflammat* AND mechanis* AND toxic* AND human – (224 hits)
   - (microplastics OR nanoplastics) AND (endocrine disrupt*) AND mechanis* AND human – (64 hits)
   - (microplastics OR nanoplastics) AND (genotox*) AND mechanis* AND human – (29 hits)
   - (microplastics OR nanoplastics) AND (apoptosis OR autophagy) AND mechanis* AND human – (155 hits)
   - (microplastics OR nanoplastics) AND (ferroptosis OR "iron dysregulation") AND mechanis* AND human – (26 hits)
   - (microplastics OR nanoplastics) AND (gut AND (microbiome OR microbiota) AND disrupt*) AND mechanis* AND human – (32 hits)
2. **Organ-specific effects:**
   - (microplastics OR nanoplastics) AND ((gut OR gastrointest*) AND toxic*) AND mechanis* AND human – 121 hits (19-07-2025)
   - (microplastics OR nanoplastics) AND respirat* AND mechanis* AND human – 81 hits (19-07-2025)
   - (microplastics OR nanoplastics) AND (cardiotox* OR "cardio* toxic*") AND mechanis* AND human – 11 hits (19-07-2025)
   - (microplastics OR nanoplastics) AND neurotoxicity AND mechanis* AND human – 67 hits (15-07-2025)
   - (microplastics OR nanoplastics) AND ("reproductive toxicity" OR reproduct*) AND mechanis* AND human – 130 hits (15-07-2025)

To minimize selection bias, we prioritized studies that (i) provided clear particle characterization (size, polymer), (ii) used appropriate controls (vehicle/leachate), and (iii) reported mechanistic endpoints. The inherent limitations of this approach are acknowledged in Section 6 of the manuscript.
